# *In vivo* transcriptomes of *Streptococcus suis* reveal genes required for niche-specific adaptation and pathogenesis

**DOI:** 10.1080/21505594.2019.1599669

**Published:** 2019-04-07

**Authors:** Jesús Arenas, Ruth Bossers-de Vries, José Harders-Westerveen, Herma Buys, Lisette M. F. Ruuls-van Stalle, Norbert Stockhofe-Zurwieden, Edoardo Zaccaria, Jan Tommassen, Jerry M. Wells, Hilde E. Smith, Astrid de Greeff

**Affiliations:** a Department of Infection Biology, Wageningen BioVeterinary Research (WBVR), Lelystad, The Netherlands; b Host Microbe Interactions, Wageningen UR, Wageningen, The Netherlands; c Department of Molecular Microbiology and Institute of Biomembranes, Utrecht University, Utrecht, The Netherlands

**Keywords:** *Streptococcus suis*, pathogenesis, zoonotic pathogen, infection, infectomics, transcriptomics

## Abstract

*Streptococcus suis* is a Gram-positive bacterium and a zoonotic pathogen residing in the nasopharynx or the gastrointestinal tract of pigs with a potential of causing life-threatening invasive disease. It is endemic in the porcine production industry worldwide, and it is also an emerging human pathogen. After invasion, the pathogen adapts to cause bacteremia and disseminates to different organs including the brain. To gain insights in this process, we infected piglets with a highly virulent strain of *S. suis*, and bacterial transcriptomes were obtained from blood and different organs (brain, joints, and heart) when animals had severe clinical symptoms of infection. Microarrays were used to determine the genome-wide transcriptional profile at different infection sites and during growth in standard growth medium *in vitro*. We observed differential expression of around 30% of the Open Reading Frames (ORFs) and infection-site specific patterns of gene expression. Genes with major changes in expression were involved in transcriptional regulation, metabolism, nutrient acquisition, stress defenses, and virulence, amongst others, and results were confirmed for a subset of selected genes using RT-qPCR. Mutants were generated in two selected genes, and the encoded proteins, i.e., NADH oxidase and MetQ, were shown to be important virulence factors in coinfection experiments and *in vitro* assays. The knowledge derived from this study regarding *S. suis* gene expression *in vivo* and identification of virulence factors is important for the development of novel diagnostic and therapeutic strategies to control *S. suis* disease.

## Introduction

*S. suis* infection in young pigs causes severe invasive disease with clinical features such as meningitis, endocarditis, pneumonia, septicaemia, and arthritis, resulting in a worldwide problem in the pig production industry. This pathogen can also infect humans and cause meningitis and other symptoms associated with septicemia [1,2]. *S. suis* infection is associated with occupational exposure to infected pigs or consumption of raw pork meat [3]. The number of human cases has increased significantly since 2005, particularly in South-East Asia where *S. suis* is now the leading cause of human meningitis [4,5]. In the *S. suis* outbreak in China in 2005, disease was more acute and severe than previously reported, causing toxic shock syndrome and high mortality [6,7]. Among the 33 capsular serotypes described in *S. suis*, serotype 2 is responsible for the majority of human and pig infections, and it is also the most commonly isolated from infected piglets worldwide [1]. Thus, the increasing emergence of severe infections and the lack of effective vaccines [8], at least against serotype 2, create an urgent need to prevent disease caused by this pathogen. Understanding *S. suis* pathogenesis and virulence will help to create effective control strategies.

*S. suis* colonizes the oropharynx and gut of pigs asymptomatically after birth [9] or through nose-to-nose contact [10]. In some animals, the pathogen is able to breach the mucosal barriers and to access and replicate in blood. Then, bacteria reach the cerebrospinal fluid, joint spaces, and serosal cavities, and rapidly invade multiple organs including spleen, liver, kidney, lung, and heart [11]. Piglets are most at risk in the period post-weaning, possibly as consequence of an immature immune system and lack of maternal immunity [11]. During colonization, invasion and systemic disease, *S. suis* encounters different microenvironments that differ, for example, in oxygen and carbon dioxide levels, concentrations of free iron, nutrient availability, or host defense factors. To ensure survival, pathogens are known to rapidly respond to environmental changes or signals in different host niches [12]. Nutrient availability in a host niche is also considered a strong driving force in the evolutionary adaptations of pathogens [13]. In several species of streptococci, including *S. suis*, a close link between carbohydrate metabolism and virulence was indeed demonstrated [14–16]. In *S. suis*, for example, carbon catabolite protein A (CcpA), regulates pathways for uptake and metabolism of carbohydrates as well as several virulence factors [14,17]. Nevertheless, much remains to be learned regarding the complete adaptation of *S. suis* during infection.

The aim of this study was to determine the whole genome expression profile of *S. suis* during infection to better understand how the bacteria adapt to different microenvironments in host tissues. Several strategies have previously been exploited to identify *S. suis* genes that are regulated *in vivo*, including *in vivo* expression technology [18], *in vivo*-induced antigen technology [19,20], or signature-tagged mutagenesis [21]. Although these approaches successfully identified individual genes involved in bacterial pathogenesis, the interdependence, orchestration and, synchronization of expression of various genes during infection was not elucidated, making it difficult to link gene expression to biological processes. To overcome these limitations, we used microarrays to study gene expression profiles of *S. suis* during an experimental infection of piglets, and thereby we analyzed characteristic sites of infection. We also aimed at validating selected genes in an independent experimental infection.

## Material and methods

### Bacterial strains and growth conditions

*S. suis* strains S735-pCOM1-*orf2* [22] and S735-pCOM1 [18], which are both derivatives of strain S735 [18] and strain 10 [23] have been described. S735 is a weakly virulent isolate of serotype 2. When *orf2* (locus tag SSU0135 in P1/7 genome) from the virulent *S. suis* strain 10 was expressed from plasmid pCOM1-*orf2* in strain S735 (S735-pCOM1-*orf2*), the virulence of the strain was increased drastically [22]. Bacterial strains were grown overnight at 37°C and 5% CO_2_ on Columbia blood base agar plates (Oxoid) containing 6% (vol/vol) horse blood and 1 µg ml^−1^ of erythromycin for plasmid maintenance. To grow the bacteria in liquid culture, one bacterial colony collected from blood base agar plates was propagated in Todd-Hewitt Broth (THB, Oxoid) supplemented with 1 µg ml^−1^ erythromycin and incubated overnight in 100-ml culture bottles in the same conditions. The culture was then diluted in THB to an optical density of 600 nm of 0.1 and incubated till exponential phase. To grow the bacteria in host body fluids, porcine serum (VWR), and heparinized fresh blood derived from 3–5 weeks old piglets (WBVR) were used. Shortly, THB cultures at logarithmic phase were diluted to ∼10^7^ colony-forming units (CFU) ml^−1^, inoculated in 100% normal porcine serum or whole blood, and incubated at 37°C. Bacterial load was monitored by CFU counting using Colombia agar plates.

### Preparation of bacterial mutants

To generate strains 10 ΔSSU0288, 10 ΔSSU0682, and 10 ΔSSU1577, chromosomal DNA of *S. suis* strain 10 was extracted and purified by standard methods (ZymoResearch) and used as template in PCR reactions. DNA segments flanking the genes of interest and a spectinomycin-resistance cassette were amplified by PCR with primers listed in supplementary Table S1. PCR assays were performed using the High Fidelity Polymerase kit (New England BioLabs) in a thermocycler. PCR products were analyzed on 1% agarose gels and purified using commercial kits (ZymoResearch). Then, PCR products (segments of flanking genes and spectinomycin-resistance cassette) were used as template for a new PCR reaction using primers flanking the extreme segments. The final PCR product was used to transform *S. suis* strain 10 following previously described procedures [24]. Transformants were selected on solid medium containing spectinomycin (100 μg ml^−1^), and the correct generation of knockout mutants was verified by PCR and sequencing.

### Animal experiments

All animal experiments performed in this study were approved by the ethical committee of the institute, in accordance with the prevailing principles and obligations of Dutch Law on animal experiments (permit numbers 2010113c and 2013033c).

#### Challenge of pigs for gene expression analysis

A total of 21 cesarean-derived colostrum-deprived (CDCD) piglets were used for transcriptomic experiments. The first 3 days of life, piglets were treated intramuscularly with enrofloxacin (Baytril®, Bayer) according to manufacturer’s instructions to prevent disease due to the general lack of passive immunity. Prior to infection, blood was extracted and collected in heparin tubes for plasma collection as well as in PAXgene tubes (PreAnalytiX) for RNA isolation. At the same time, tonsils of all piglets were sampled with swabs, and the absence of colonization by *S. suis* was verified by PCR analysis [23] using specific primers for the *S. suis gdh* gene [25] (supplementary Table S1). At the age of 4 weeks, the animals were infected intravenously on study day 0 with 10^6^ CFU of S735-pCOM1-*orf2* (*n* *=* 15) or S735-pCOM1 (*n* *=* 6). After infection all piglets were orally administered erythromycin (40 mg kg^−1^ body weight) twice daily to keep selection pressure on plasmids. Blood was collected in heparin tubes and PAXgene tubes (Qiagen Hilden) at days 1, 2, 3, and 4 postinfection. Rectal temperatures and health status of piglets were recorded four times a day, scoring nonspecific (reduction in appetite and depressive behavior) and specific symptoms [lameness, central nervous system (CNS) symptoms (i.e. locomotor disorders, opisthotonos, and nystagmus), kyphosis (arched back) and shivering, the latter two being considered as symptoms of sepsis and serositis] of streptococcal disease. When piglets showed specific symptoms of *S. suis* infection, they were euthanized to minimize suffering, otherwise they were euthanized at the end of the experiment (6 days postinfection). During necropsy, tissues known to be affected by *S. suis* infection were examined carefully, i.e. various joints, peritoneal, and pleural cavity, pericardia and CNS specimens were taken to determine the presence of *S. suis* by CFU counts. Tissue samples (CNS, joint capsule, heart, lungs, and spleen) were also fixed in 4% of formaldehyde for histology. For pathohistological analysis, formalin-fixed organs were embedded in paraffin, sectioned at 3–5 μm, and slides were stained with hematoxylin and eosin and examined for the presence and the degree of inflammation. “Mean survival postinfection” was calculated by averaging the survival time in days from inoculation until they showed specific symptoms of *S. suis* infection and used as a measure of disease severity. “Fever Index” was calculated as a percentage of the number of observations in which piglets had a body temperature ≥40°C divided by the total number of observations.

#### Coinfection experiments

To assess the contribution of specific genes to infection, coinfection experiments were performed. Four-week-old piglets from a farm free of relevant swine pathogens and without history of *S. suis* were used. Piglets were confirmed to be free of *S. suis* infections by PCR examination of tonsil swabs. Piglets were infected intravenously with 10^6^ CFU of strain 10 and 10^6^ CFU of an isogenic mutant. Blood samples were collected on a daily base to monitor for bacterial growth. As soon as animals showed specific signs for *S. suis* infection as described above, animals were euthanized and subjected to necropsy as described. Organs of infected animals were weighed, mixed with one volume of THB medium and homogenized in a Stomacher 400 (Seward). The ratio of the mutants over wild-type bacteria in blood, serosa fluids, joint fluids, and homogenized organs were determined by plating on Colombia agar, either containing or not spectinomycin, and CFU counting after overnight incubation. Data obtained from animal experiments were analyzed with GraphPad Prism version 7 for statistical comparisons using unpaired t test.

### Bacterial RNA isolation from standard laboratory cultures and infected pigs

To obtain RNA from *in vitro* cultures, cells from cultures growing in THB, at exponential and stationary phases, were harvested by centrifugation (20,000 *x g* during 10 min), and the resultant pellet was snap-frozen in liquid nitrogen. Triplicate RNA extractions were performed on two independent cultures as described below.

To extract bacterial RNA from different infection sites, bacteria in fluids of inflamed joints, pericardial fluids from heart and from the surface of the meninges were collected by rinsing the organs with sterile PBS. The selected sites showed signs of inflammation and massive bacteria loads and were therefore optimal for RNA extraction. All collected fluids were diluted 1:1 with PBS, centrifuged for 5 min at 850 *x g* to remove host cells, and bacterial cells were harvested by centrifugation (20,000 *x g* 10 min). The resulting pellets were snap-frozen in liquid nitrogen. A total of 21 bacterial pellets isolated from 10 individual piglets were collected (Table S2 in supplemental material). The frozen pellets were thawed on ice, suspended in 600 µl of Trizol (Invitrogen), and bacteria were disrupted during 40 s in a Fastprep-24 homogenizer (MP Biomedicals). Then, 120 µl of chloroform was added. After mixing for 15 s, the mixture incubated for 3 min at room temperature (RT) and then centrifuged for 15 min at 20,000 *x g*. The resulting supernatant was mixed with 1 volume of chloroform, and RNA was precipitated by adding 1 volume of isopropanol and incubation for 30 min at RT or for 16 h at −20°C. RNA was then collected by centrifugation, washed with 70% ethanol and suspended in water. Subsequently, RNA was purified using a Nucleospin RNA II kit (Macherey-Nagel) according to manufacturer’s instructions. Eukaryotic RNA was removed from *in vivo* samples using the MicrobEnrich kit (Ambion).

### Reverse transcription—Quantitative PCR (RT-qPCR)

About 20–50 ng of bacterial RNA was used to generate cDNA using the Ovation PicoSl WTA system v2 (Nugen) following manufacturer’s indications, and the resulting cDNA was purified with MinElute spin columns (Qiagen). Fragments of selected *S. suis* genes were amplified by qPCR in an ABI7500 system (Applied Biosystems) using purified bacterial cDNA as template. Included were cDNA preparations generated from bacterial RNA extracted from different sites of infected animals (supplementary Table S2) and from bacteria growing in THB medium. The qPCR reactions were done in tryplo with primer pairs listed in supplementary Table S1 and POWR SYBR Green PCR Master Mix (Applied Biosystems) according to manufacturer’s instructions. GeNorm identified *gyrA, proS*, and *mutS* as the most stably expressed reference genes (*n* = 7) for normalization of all data using the ∆ -Ct method [26]. For each PCR reaction, a standard curve consisting of tenfold serial dilutions of a vector containing the gene of interest or water as negative control were incorporated. Data analysis was performed using the ABI7500 Software (Applied Biosystems).

### Microarray analysis

cDNA preparations that were positive for *S. suis orf2* by qPCR were used for microarray experiments. cDNA (0.5–2.5 µg) was labeled with cyanine 3-d-UTP using the Genomic DNA Enzymatic Labeling kit (Agilent Technologies) according to manufacturer’s instructions and purified using Amicon Ultra-30K membrane filters (Millipore). Prior to hybridization, pure labeled cDNA in Agilent blocking reagent and Agilent Hi-RPM buffer was incubated for 3 min at 95°C followed by 30 min at 37°C. The mixture was then hybridized to one array of an 8 × 15 k custom *S. suis* oligo-array (Agilent Technologies) containing probes based on genome sequences of strain P1/7 [27] and probes designed on incomplete genome sequences of strains 891,591, 8067, 7917, and 6388 and incubated at 65°C in a rotator hybridization oven. After 17 h, arrays were washed for 5 min at RT in Oligo aCGH wash buffer 1 (Agilent Technologies) and subsequently for 1 min at 37°C in Oligo aCGH wash buffer 2. Arrays were dried at RT and scanned using a Surescan high-resolution DNA microarray scanner (Agilent Technologies), followed by data analysis using Feature extraction software and Genespring GX software, both also from Agilent Technologies.

### Bioinformatics

Data derived from microarrays were clustered in Genespring to determine similarity of samples using hierarchical clustering based on the Pearson correlation coefficient, and linkage was determined using the average neighbor’s method. KegArray software (available at http://www.genome.jp/kegg/) was used for pathway analysis of regulated genes, and gene ontology was attributed from annotated predictions in publicly available *S. suis* genome sequences.

## Results and discussion

### *Experimental infection of piglets with S735-pCOM1-*orf2 *strain causes streptococcal disease*

*S. suis* strain 10 and P1/7 are clinical isolates of serotype 2 and used as reference strains for pig infection experiments. Therefore, they are well suited for transcriptomic studies *in vivo*. However, to reduce the number of animals, we used *S. suis* strain S735-pCOM1-*orf2* [22]. Strain S735-pCOM1-*orf2* is more virulent than strains 10 and P1/7, and it can be massively recovered from inner organs and blood in experimental animal infections. Thus, 15 piglets were infected intravenously with the *S. suis* strain S735-pCOM1-*orf2*. All piglets developed severe invasive infections within 2 days with a mean fever index of 48% and mean survival of 51 h postinfection. Table 1 summarizes the clinical symptoms, bacteriology, and histopathological findings in piglets after experimental infection. *S. suis* was isolated from CNS of 12 piglets, seven of which developed clinical meningitis. Additionally, arthritis was apparent in 14 piglets, pericarditis in 11 piglets, and pneumonia in 13 piglets. Severe inflammation of serous membranes (pleura, pericardium, and peritoneum) was observed within 60 h postinfection (data not shown). Bacteriological examination at different sites of infection confirmed that indeed all piglets were infected with *S. suis*. The pathogen was isolated from 141 out of 225 specific sites of infection (63%), including joints, heart, and brain (Table 1). To verify that the induced clinical symptoms were characteristic of *S. suis* infections, histopathological studies were conducted on different organs. Examination of cerebrum, cerebellum, and brain revealed a fibrinpurulent meningitis in 10 out of the 15 piglets (Table 1), indicating typical acute infection. Incidentally, encephalitis was observed. In the synovia of 10 out of 15 piglets a fibrinopurulent with microvascular thrombi and fibrinopurulent epicarditis with large influx of neutrophils was found. All six piglets infected with the *S. suis* isolate S735-pCOM1 as a control survived till the end of the experiment, and in general they showed nonspecific symptoms of streptococcal infections (indicated in material and methods). In conclusion, *S. suis* strain S735-pCOM1-*orf2* induced a very acute onset of streptococcal disease symptoms as previously described [6].

**Table 1.** Clinical symptoms, bacteriology, and histopathology in 15 piglets after experimental infection with *S. suis* strain S735-pCOM-*orf2.*

Gross pathology	Meningitis No. of animals	Arthritis No. of animals (No. of joints)	Pericarditis No. of animals	Pneumonia No. of animals
	7	14 (46)	11	13
Bacteriology	**Cerebrospinal fluid** No. of animals	**Joints** No. of joints	**Heart** No. of animals	**Lungs** No. of animals
	12	105/180	13	11
Histopathology (meningitis)	**Cerebrum** No. of animals	**Cerebellum** No. of animals	**Brain stem** No. of animals	
	10	10	10	

The numbers of affected animals and joints and the total of examined joints are indicated.

### Global changes in the transcriptomics during invasive infection

**Figure 1.**
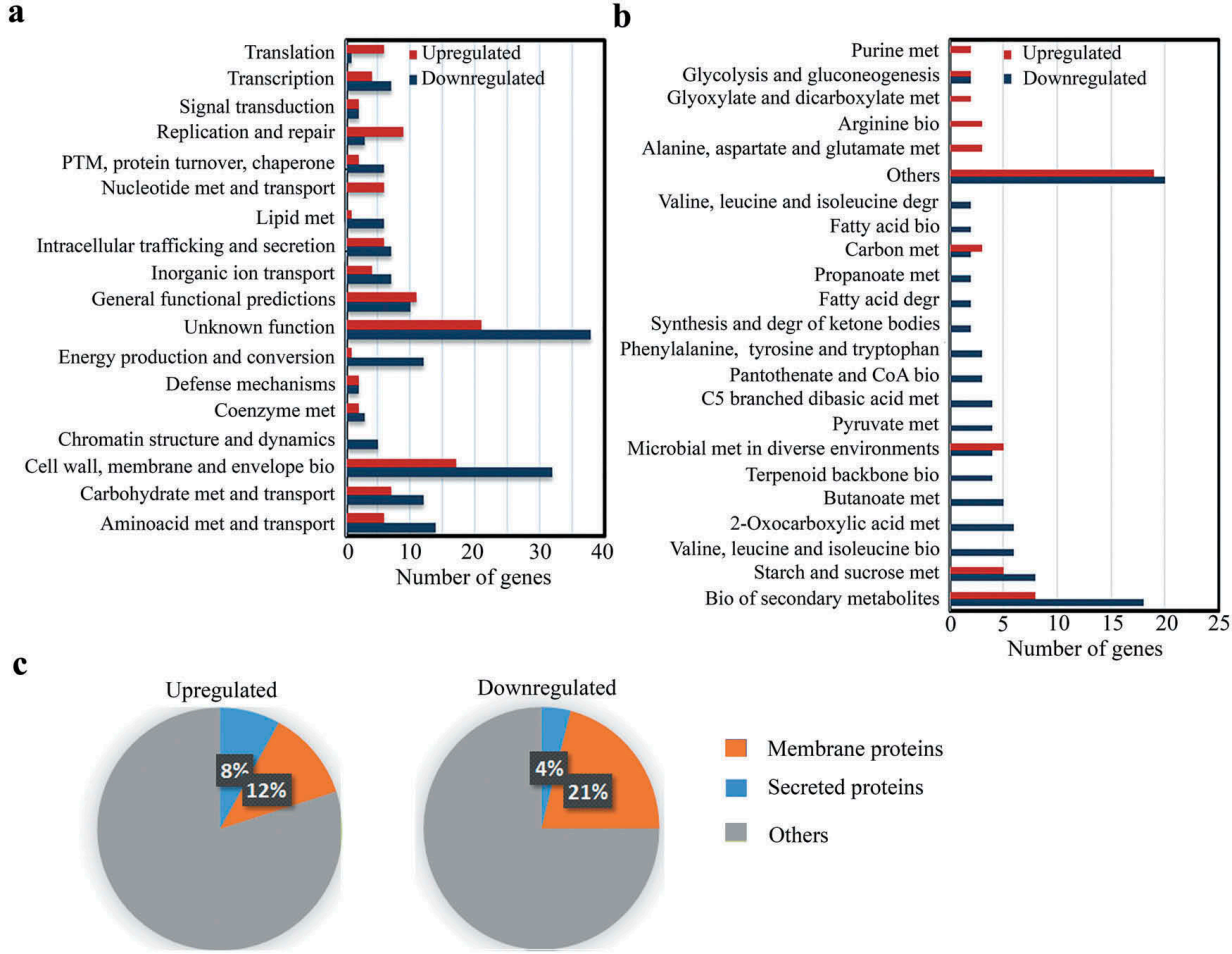
Classes of genes differently expressed in *S. suis* during infection vs growth in culture medium. The numbers of up- and downregulated genes clustered according to (a) annotated COG categories and (b) metabolic pathways based on KEGG database is given, as well as (c) the predicted localization of the corresponding gene products. Only significantly expressed genes homologous to genes found in the P1/7 genome with at least 2 log_2_ FC differences and statistical significance *p* < 0.01 were considered. In panel (b), categories with only one gene were not considered and classified as others. Abbreviations: PTM: post-translational modifications; met: metabolism; bio: biosynthesis; degr: degradation.

Genome-wide transcription profiles of strain S735-pCOM1-*orf2* isolated from blood and from infection sites that showed large signs of inflammation during experimental infection [i.e. meningeal fluid (hereafter brain), joint fluid (hereafter joint), and pericardial fluid (hereafter heart)], and from logarithmic THB cultures were obtained as described in section “Materials and methods.” All genes differentially expressed *in vivo* from all infection sites (average) compared to *in vitro* are listed in supplementary Table S3 along with *p* values and fold change (FC) differences, assigned gene features and homologies. A total of 665 genes on the array, i.e. about 30% of the genome, was differentially transcribed (*p* < 0.01). Genes were further ranked based on an FC of at least two (log_2_ ratio ≤−2 and ≥2) and on the presence of a homologue in the publicly available P1/7 genome sequence, resulting in 257 genes. Selected genes were then grouped according to biological processes, molecular functions, and cellular localization (Figure 1). According to assigned gene ontology annotations, a large number of genes that were differently regulated *in vivo* had no known predicted function (22% of annotated genes) or a general-function prediction (5.7% of annotated genes) (Figure 1(a)). Some of those genes are organized in operons. Examples are two operons, each containing a group of genes (SSU1277-SSU1280 and SSU1551-SSU1556) encoding predicted membrane proteins of unknown function, which are strongly downregulated *in vivo* compared to *in vitro*. Of the 257 differentially regulated genes, 18% are involved in cell wall, membrane, and envelope biogenesis, 8.1% in amino-acid metabolism and transport, 6.9% in carbohydrate metabolism or transport, and 6.9% in energy production or conversion, among others (detailed in Figure 1(a)). In most of these functional categories, the majority of the genes were downregulated during infection. Exceptions are the functional categories translation, replication and repair, and nucleotide metabolism and transport, which mostly contain upregulated genes (Figure 1(a)). For specific biosynthesis and degradation pathways of certain amino acids, fatty acids, ketone bodies, terpenoid backbone or pyruvate metabolism, among others, all genes were either up- or downregulated (Figure 1(b)). In contrast, several general pathways, such as biosynthesis of secondary metabolites, starch, and sucrose metabolism, carbon metabolism or glycolysis and gluconeogenesis, included both up- and downregulated genes (Figure 1(b)). Besides, 22 genes encoding hypothetical transporters for sugars, metals, amino acids, and vitamins, among others, were also regulated, of which 15 and 7 were down- and upregulated, respectively. Many genes coding for predicted integral membrane proteins were up- (12%) or downregulated (21%) (Figure 1(c)). A lower number of genes encoding secreted proteins, including lipoproteins and surface-anchored proteins, was also identified, of which 8% and 4% were up- and downregulated, respectively (Figure 1(c)). Overall, although many genes were constitutively expressed, these data reflect large differences in expression of genes belonging to a variety of functional categories when comparing *S. suis* grown in the host vs. THB medium.

### Niche specific expression patterns during infection

**Figure 2.**
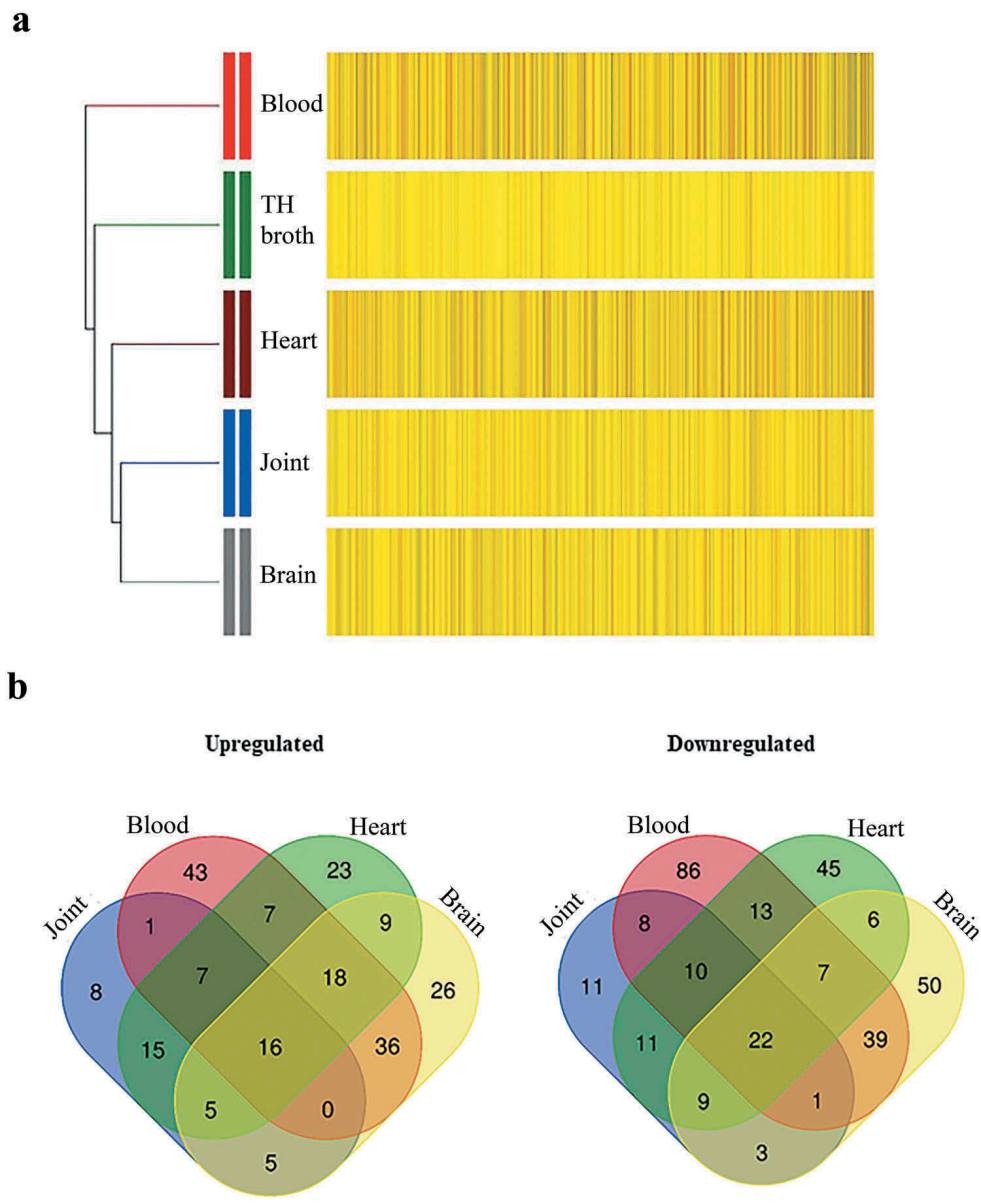
Profiles of up- and downregulated genes at different sites of infection. (a) Hierarchical clustering of the differentially expressed genes showing the data for all indicated infection sites and for growth in liquid culture (TH broth) at stationary phase. Clustering shows well-defined partitions for each site of infection. For all groups, upregulated (yellow) and downregulated (black) genes are indicated as relative to THB cultures at logarithmic phase. (b) VENN diagram indicating the numbers of regulated genes at each infection site. Genes were selected based on an FC of at least two (log_2_ ratio ≤−2 or ≥2) and a *p* < 0.01. Sites of infection were meningeal fluid (indicated as brain), joint fluid (indicated as joint), pericardial fluid (indicated as heart) and blood.

We next investigated bacterial gene expression for each infection site compared to THB medium. Table S4 in supplemental material lists the complete dataset of regulated genes (*p* < 0.01) for each site of infection, including putative or demonstrated functions, *p* values and FC differences. The expression profiles of the 552 selected genes were clustered by hierarchical clustering analysis (Figure 2(a)). Transcriptome profiles derived from organs and THB were similar and distinct from those derived from bacteria recovered from blood. This is in accordance with a large number of genes differentially expressed in blood (*n* *=* 406), followed by brain (*n* = 261), heart (*n* = 222), and joints (*n* = 111) compared to *in vitro* cultures. Remarkably, 300 genes were exclusively altered in one infection site, mostly in blood. Figure 2(b) details the numbers of up- and downregulated genes within infection sites for filtered genes (FC log_2_ ratio ≤−2 and ≥2). Expression of many genes (*n* *=* 248) was altered at least in two infection sites, and some genes (*n* = 38) were altered in all sites of infection. A total of 26 genes revealed no significant differences in expression when sites of infection were compared to each other. These data are suggestive of specific transcriptome profiles associated with each infection site. In summary, our comparisons indicate that *S. suis* adapts gene expression for dissemination in the host.

To validate the transcriptomics data obtained by microarrays, the expression profiles of a subset of representative genes were analyzed by RT-qPCR. We selected genes with variable expression level at different infection sites and from different functional categories, i.e. SSU0357, SSU0899, and SSU1849. Figure S1 (supplemental material) shows gene expression profiles obtained by RT-qPCR and microarrays for each selected gene. Except for gene SSU0899, where transcription levels in blood and joint were upregulated in RT-qPCR but not in microarrays, RT-qPCR analysis confirmed our microarray data.

### Involvement of regulators in the adaptation of S. suis to the host

**Figure 3.**
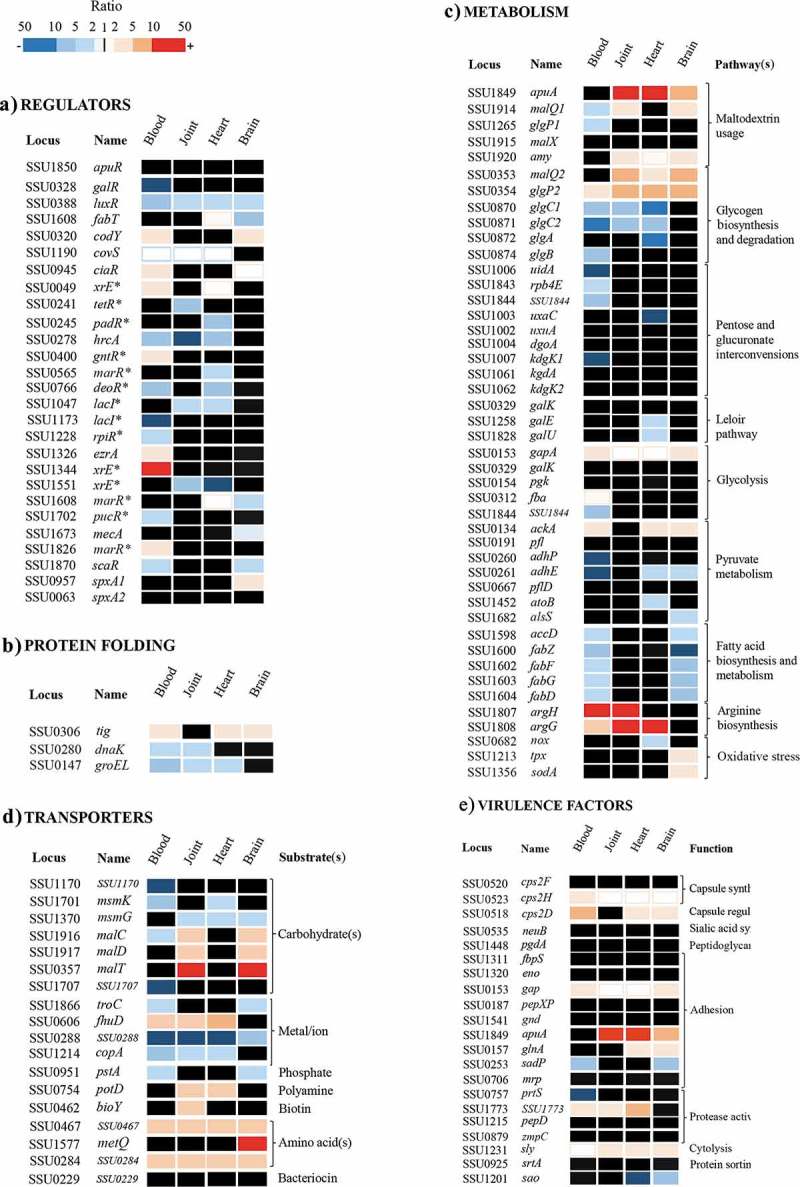
Heatmap showing variable regulation of genes participating in diverse functional categories at different sites of infection. The genes depicted are involved in the functional categories regulators (a), protein folding (b), metabolism (c), transporters (d), and virulence factors (e). Sites of infection are indicated at the top of each panel. The corresponding locus tag in the P1/7 genome, gene name (when appropriate), and predicted or demonstrated pathway (panel c), substrates (panel d), and functions (panel e) are depicted. Gene names according to the annotated P1/7 genome sequence or the literature are provided. Alternatively, names with one asterisk refer to regulator family type. The gene expression levels obtained in the microarray studies are represented in a color scale (indicated at the top) according to FC *in vivo* vs. THB growth cultures at logarithmic phase. Red indicates induction and blue repression. Whereas appropriated, unregulated genes are also shown. The figure pretends to be representative rather than exhaustive; for an extensive list, see supplementary Table S4.

A total of 35 genes encoding for putative regulators were differently expressed when bacterial transcriptomes derived from infection sites were compared with those from bacteria grown *in vitro* in THB (see Figure 3(a) for examples). The number of differentially expressed regulators varied considerably for bacteria from blood and tissues (20 for blood, 7 for joint, 12 for heart, and 9 for brain). Thus, in the transition from THB via blood to tissues, the expression of many regulators is first changed in blood and it is changed again in the transition from blood to tissues, in line with the transcription profiles (Figure 2(a)).

Many of the identified regulators have key roles in metabolism and transport that can be also related to pathogenesis and virulence. Among them, the global regulators CodY and CiaR that were upregulated in the bacteria from blood and brain (Figure 3(a)). CodY controls gene expression in response to amino acid availability and stress conditions [28]. A *codY* mutant of an *S. suis* serotype 2 strain was attenuated in a mouse infection model and significantly inhibited in resistance to phagocytosis [29]. CiaR is part of the CiaHR two-component system, which is widely distributed among streptococci [30]. In *S. suis*, CiaRH system mutants showed increased susceptibility to killing by macrophages and were also attenuated in mice and pig models [31]. Also in a group B *Streptococcus*, a *ciaR* mutant showed reduced survival in neutrophils, human macrophages, and brain microvascular endothelial cells [32]. Hence, we speculate that the *S. suis* regulators CodY and CiaR may promote resistance to phagocyte clearance and antimicrobial peptides in blood and/or meninges, which is key to survival of the pathogen in the bloodstream, for blood-brain barrier penetration, and for colonization of the meninges.

Transcripts of *covS*, the product of which is part of the CovRS regulatory system, were slightly downregulated at several sites of infection as compared to THB (Figure 3(a)). CovRS is a global repressor of virulence and colonization in many pathogenic bacteria. Together with the upregulation of *codY* and *ciaR*, this indicates that *S. suis* requires different virulence factors to survive in particular infection sites. Other differentially expressed regulators play a role in responses to stress conditions. Examples include the HrcA and SpxA1 proteins. The *hrcA* gene was downregulated at all sites of infection except brain (Figure 3(a)). HrcA is a repressor protein that controls the expression of the genes of DnaK and GroEL operons. These chaperones participate in the folding of nascent proteins, refolding of damaged or misfolded proteins, protein secretion, and presentation of damaged proteins to degradative pathways [33,34]. Unexpected, *dnaK* and *groEL* were downregulated (Figure 3(b)) whilst, in contrast, the *tig* gene that codes for the chaperone trigger factor was upregulated at diverse sites of infection (Figure 3(b)). Deletion of *tig* in the *S. suis* strain sc21 resulted in enhanced transcript levels of *hrcA in vitro* experiments [35], which could partially explain downregulation of *hrcA* at certain infection sites. In any case, this suggests that the production of some chaperones could be regulated *in vivo* by multiple factors. In contrast to *hrcA, spxA1* was specifically upregulated in brain tissues (Figure 3(a)). SpxA1 proteins upregulate several *S. suis* genes known to be involved in oxidative stress responses [36], such as *nox, tpx,* and *sodA*, which encodes a NADH oxidase, a thiol peroxidase and a superoxide dismutase, respectively. Indeed, *tpx* and *sodA*, but not *nox*, were upregulated in brain tissues (Figure 3(c)). Hence, the differential regulation of *hrcA* and *spxA* reflects different counteracting responses of the pathogen to environmental stresses imposed by the host in particular infection sites. In addition, expression of the genes for several other regulators of diverse families, such as MarR, XRE, PadR, TetR, and GntR, was differentially altered (Figure 3(a)); yet, their particular role during *S. suis* infection remains to be investigated. Overall, these data indicate that *S. suis* requires a specific repertoire of regulators to adapt to the different organs and blood during infection.

### Adaptation of S. suis metabolism during infection

The expression of genes involved in metabolic pathways and transport systems varied for bacterial transcriptomes isolated from different organs and blood, in accordance with the variety of genes encoding for different regulators. Considering the rapid onset of the disease and the variable composition of nutrients in body sites, this indicates a rapid adaptation of the bacterial metabolism when the pathogen gains access to blood and disseminates to organs. The ability of *S. suis* to use different carbohydrates plays an important role in survival during infection or colonization, and it has been related with virulence [14]. The alteration of expression of representative genes coding for enzymes of different metabolic pathways is illustrated in Figure 3(c) and summarized in Table S5 (supplemental material). Several genes of the pathway for the utilization of maltodextrin and maltodextrin-containing polymers were upregulated in tissues but not in blood (Figure 3(c)). Among these genes are *apuA* and *amy*, encoding an amylopullulanase and an α-amylase, respectively. These enzymes cleave α-1,4 and α-1,6 glycosidic bonds between glucose residues in starch or mammalian glycogen, resulting in partially hydrolyzed structures like maltodextrins, disaccharides, and trisaccharides. Expression of *apuA* has been shown to be downregulated in culture medium in the presence of glucose [14], which is relatively abundant in blood (4.4–6.6 mM) compared to THB medium, and upregulated in the presence of pullulan, a starch, which is present in the intestine, oral cavity and in different tissues (joint, brain, and heart) (0.25–0.55 mM). The upregulation of the genes for these hydrolytic enzymes is in accordance with the upregulation of the *malC* and *malD* genes encoding components of a putative ABC transporter for maltose and maltodextrins and of *malT* encoding a component of a putative bifunctional PTS transporter for glucose and maltose (Figure 3(d)). Internal glycogen is degraded to glucose 1-phosphate by GlgP2 and MalQ2 and synthetized from glucose1-phosphate by GlgC, GlgA, and GlgB. The corresponding genes are organized in an operon. Whilst genes encoding proteins that participate in glycogen degradation were upregulated, genes for proteins involved in glycogen biosynthesis were downregulated in blood, joints, and heart but not in the brain (Figure 3(c)), suggesting the necessity for *S. suis* to utilize glycogen rather than to store it. *Streptococcus* spp. can also use lactose and galactose as energy sources through the tagatose 6-phosphate pathway, and galactose through the Leloir pathway. In *S. suis* isolated from heart tissue, genes encoding enzymes that participate in either of the above pathways were downregulated, e.g. the *galU* and *galE* genes of the Leloir pathway (Figure 3(c)). These enzymes produce UDP-glucose and UDP-galactose, respectively. Both are precursors for different virulence factors such extracellular polysaccharides. Indeed, *galU* expression was shown to be essential for capsule biosynthesis in *S. pneumoniae* [37] and upregulation of the Leloir pathway through the autoinducer AI2 led to increased production of capsular polysaccharide with a subsequent hypervirulent phenotype [38]. As the capsule is also considered an important virulent factor in *S. suis* [39–44], why would *S. suis* downregulate capsule production in heart tissues? It has been reported that 34% of *S. suis* isolates from cases of porcine endocarditis lost their capsules, whilst, in contrast, 100% of meningitis isolates that were capsulated [45]. Loss of capsule enhances the ability of the bacteria to adhere to porcine cells and to generate thick biofilms, thus facilitating endocarditis [46,47]. It is, however, important to mention that we extracted bacterial RNA from pericardium-associated bacteria rather than from endocardium. Thus, apparently, downregulation of genes involved in the Leloir pathway could also be relevant for colonization of the pericardium.

*Streptococcus* spp. metabolize sugars into pyruvate and convert pyruvate into lactate or into formate and acetyl-CoA. Acetyl CoA is the substrate in the pathway for fatty acid biosynthesis. Interestingly, many genes that participate in the fatty acid metabolism and biosynthesis pathways, some of which are organized in an operon comprising SSU1596-SSU1609, were downregulated in blood and brain (supplementary Table S5). Fatty acids are rather abundant in serum, blood, and brain, and it has been reported that several Gram-positive bacteria can incorporate exogenous fatty acids in their membranes [48,49]. On the other hand, alteration of the fatty acid synthesis pathway leads to variation in the membrane composition that may contribute to resistance to antimicrobial peptides and lytic enzymes produced by the host [50]. It has been shown that a cathelicidin-related antimicrobial peptide is produced in the endothelial cells of the blood-brain barrier and in cells of the meninges after meningococcal infection of mice [51]. Thus, low requirements for fatty acid biosynthesis could be related with resistance to host defenses. In addition, regulation of genes involved in the biosynthesis of amino acids was specifically associated with the sites of infection, and this was also observed for genes involved in many metabolic routes (summarized in supplementary Table S5).

The different availability of nutrients for *S. suis* in diverse body sites with the consequent activation of a variety of metabolic pathways also resulted in the altered expression of many genes (*n* *=* 47) that code for (components of) putative transporters. Examples include the *malC* and *malD* genes encoding components of an ABC transporter of maltose and maltodextrins, and PotD, the substrate-binding component of an ABC transporter of polyamines (Figure 3(d)). A total of 13 genes encoding components of 11 transporters was exclusively altered in one infection site. The genes *metQ* or *bioY*, which encode substrate-binding proteins for methionine and biotin (Figure 3(d)), respectively, are examples. Some transporters are assigned functions in metal transport, reflecting the nutritional immunity imposed by the host. The gene encoding FhuD, the substrate-binding lipoprotein of an ABC transporter involved in iron acquisition [52], was highly upregulated in all infection sites, with the exception of brain (Figure 3(d)). Sera derived from mice infected with *S. suis* contained antibodies against this protein [53], confirming that it is indeed expressed *in vivo*. Genes coding for a putative cobalt transport system (SSU0264-SSU0267) were upregulated only in joint and heart. In contrast, genes corresponding to other metal-transport systems were downregulated including those for a zinc/manganese transporter (SSU1865-SSU1867), which were downregulated in blood and brain, and for two putative heavy metal-transporting P-type ATPases (SSU0288 and SSU1214). The substrates of these latter transporters may include several metal ions, e.g. copper (SSU1214), and in most cases, they act as efflux pumps of toxic metal ions [54]. The strong downregulation of the genes encoding these transporters most likely reflects a lower abundance of these ions *in vivo* than *in vitro.*

### Regulation of putative and known virulence factors

Of the 26 genes encoding known or putative virulence factors investigated, 10 were differentially regulated in expression (Figure 3(e)). Among them, several genes encoded for extracellular proteases. One of them, SSU1773, which was upregulated in bacteria from blood, joint, heart, but not in brain, as compared to THB-grown bacteria. The function of this protein has not been elucidated. Sequence alignments of the predicted protein revealed that it is member of the subtilisin-like serine protease family. The protein contains an N-terminal signal peptide with a YSIRKxxxGxxS motif, required for protein secretion and processing. The C-terminal extreme contains a LPXTG motif for anchoring of the protein to the bacterial cell wall. The signal peptide is followed by a peptidase S8 family domain, which is interrupted by the insertion of a PA domain in a loop, and an fn3_5 domain, frequently found in Streptococcal C5a peptidases [55]. C5a peptidases cleave the complement component C5a, which is key to neutrophil activation and recruitment to the site of infection. In addition, these proteins can function as adhesins/invasins to epithelial and endothelial cells [56,57]. Indeed, SSU1773 was identified as relevant for adherence to host cells in a recent screening of TraDIS libraries of *S. suis* P1/7 *in vitro* organ culture system [58]. However, the protein lacks a conserved RGD motif usually involved in integrin binding. Furthermore, the recombinant protein reacted with sera of convalescent pigs [58] showing that it is produced during infection, in line with our observations. Interestingly, immunization with this protein in combination with four other antigens elicited a protective response against *S. suis* in pigs after an intranasal challenge [58]. Together, these works reflect the potential role of SSU1773 in the colonization of the upper respiratory tract and in pathogenesis. In contrast to SSU1773, we did not find altered expression of the *zmpC* gene encoding a surface-anchored zinc metalloprotease C *in vivo*. Earlier studies showed that a recombinant ZmpC of *S. suis* strain 05ZYS cleaves human IgA1 [59], and a *zmpC* mutant was attenuated in a pig infection model [60]. However, this activity has been recently disputed, as *S. suis* strains P1/7, CCUG7986, CCUG7984, and CCUG42755, which contain a *zmpC* gene, do not cleave human IgA1 [61]. In line with this notion is that *S. suis* ZmpC shares structural homologies with *S. pneumoniae* ZmpC that lacks IgA1 protease activity [62]. Pneumococcal ZmpC activates the matrix metalloproteinase 9 and cleaves P-selectin glycoprotein ligand-1, mucin 16 (MUC16), and syndecan-1 (SDC-1) ectodomains, while *S. suis* ZmpC has only a modest activity on MUC16 and SDC-1 [61]. The absence of this activity in a *zmpC* mutant did not drastically affect the ability of *S. suis* to colonize the upper respiratory tract of pigs [61]. Possibly, the absence of *zmpC* regulation in blood and tissues observed in our study may also reflect the absence of a crucial function during organ colonization, sepsis, and meningitis, at least in *S. suis* strain S735-pCOM1-*orf2*.

We detected high expression levels in tissue-associated bacteria of the *sly* gene that codes for suilysin. Suilysin is a secreted extracellular protein belonging to the family of cholesterol-dependent cytolysins that form transmembrane pores on different eukaryotic cells. Expression of *sly* was slightly increased in blood as compared to THB medium (Figure 3(e)) in accordance with its hemolytic capacity [63], which results in the release of hemoglobin. Hemoglobin increases the release of several proinflammatory mediators (IL-1β, TNF-α, IL-6, and IL-8) by macrophages by acting in synergy with cell-wall components of *S. suis* [64]. In addition, suilysin reduces phagocytosis and killing of *S. suis* independent of its cytotoxicity [65], and it induces the formation of platelet-neutrophil complexes that are involved in inflammation and organ failure in some bacterial infections [66]. Expression of *sly* was higher upregulated in tissue-associated bacteria than in blood. One possible explanation for this is that suilysin causes cytotoxicity to epithelial cells [67] resulting in the release of glycogen in infected organs, which can then be enzymatically degraded by the *apuA*-encoded amylopullulanase into maltodextrins to be transported and metabolized by the pathogen. Moreover, suilysin is toxic for the main cellular type of the blood-brain barrier, i.e. microvascular endothelial cells. This is consistent with the upregulation of *sly* in meninges-associated bacteria in our study.

A subset of genes that encodes surface-exposed proteins with functions in adhesion were not regulated *in vivo*. For example, *eno* that codes for enolase. Enolase binds plasminogen and fibrinogen [68] and it was suggested that it helps the pathogen to cross the blood-brain barrier and gain access to the CNS [69]. Also, by interacting with fibrinogen, enolase inhibits phagocytosis and enhances *S. suis* survival in blood [70]. Similarly, we did not find upregulation of the *fbpS* gene, which also encodes fibrinogen-binding protein, in bacteria recovered from blood or host tissues. A *fbpS* mutant resulted attenuated in a competitive infection model in piglets [71]. *mrp* that encodes a muramidase-release protein (MRP) with fibrinogen binding properties was also not regulated. Like enolase, MRP is often associated with virulence and reported as a marker for highly virulent strains of Europe and Asia. Albeit expression of *eno, fbpS,* and *mrp* was related to virulence, alternative works suggest the dispensability of some of these factors for *S. suis* virulence [reviewed in 72], which may explain the lack of regulation in our work. In contrast to these adhesins, genes that code for several intracellular enzymes reported to be involved in adhesion were regulated (Figure 3(e)). Some of these enzymes were shown to be located at the bacterial surface, although they lack a signal peptide required for secretion. For instance, *gap* that codes for glyceraldehyde-3-phosphate dehydrogenase and was upregulated at several infection sites (Figure 3(e)). This enzyme was shown to contribute to adherence in different Streptococcus species, including *S. suis* [73], and to resistance to phagocytosis in group A and B Streptococcus [74]. The enzyme was located at the cell surface of Group B Streptococcus, and proposed to be released upon cell lysis and then associated with bacterial surface whereas exerts its function as a adhesion. Other example is *glnA* that was upregulated in heart and brain tissues. *glnA* encodes a glutamine synthetase involved in the formation of glutamine from glutamate and ammonia at the expense of ATP and this function affects adhesion to epithelial cell lines [75]. Importantly, addition of exogenous glutamine to a *glnA* mutant restored the loss of adhesive properties of *S. suis* [75], and thus, in contrast to glyceraldehyde-3-phosphate dehydrogenase, the role of glutamine synthetase on adhesion might be caused by an altered glutamine metabolism. In any case, upregulation of *glnA* expression in heart and brain tissues is in partial agreement with previous results of colonization experiments in mice models showing that a *glnA* mutant displayed a significantly reduced number of bacteria in heart and brain [75]. Yet, this could be explained by glutamine requirements under these conditions rather than putative adhesive functions. Moreover, the gene encoding the surface antigen one (Sao) was downregulated in heart and brain as compared to blood and joints (Figure 3(e)). Sao is an immunogenic protein and a protective antigen [76], but the contribution of Sao to *S. suis* virulence remains controversial [77].

Capsule is universally considered as a virulence factor for *S. suis* [39–44]. It inhibits phagocytosis by neutrophils and macrophages [39], and isogenic unencapsulated mutants were attenuated for virulence in pig and mouse models [43,44]. On the other hand, the absence of capsule facilitates the interaction of *S. suis* with different cell types [11,78]. Hence, it has been suggested that capsule synthesis must be regulated to facilitate tissue colonization and interbacterial interactions during the colonization and invasion process. Of all genes encoding proteins for capsule biosynthesis (*cps2E-N*) and polymerization (*cps2*I), only *cps2H* was upregulated in transcriptomes derived from blood as compared to growth in THB (Figure 3(d)). *cps2H* encodes a glycosyl transferase that participates in the formation of the repeat unit by linking galactose to rhamnose. However, upregulation of only one enzyme does not necessarily yield higher capsule production, but this is possible if this enzyme catalyze the rate-limiting step. Alternatively, the thickness of the capsule can be regulated by a phosphoregulatory system encoded by *cps2A, cps2B, cps2C*, and *cps2D*. In *S. pneumoniae*, CpsC and CpsD constitute the integral membrane activation domain and the cytoplasmic kinase domain of an autophosphorylating tyrosine kinase. CpsB (encoded by *wzh* or *cps2D* in *S. suis*) is a manganese-dependent phosphatase capable of dephosphorylating phosphorylated CpsC [79]. In our study, *cps2D* was upregulated when *S. suis* infected blood, brain, and heart tissues (Figure 3(a)). Deletion of *cps2B* in *S. pneumoniae* resulted in increased phosphorylation of CpsD, but the resulting capsule phenotype varied [79,80]. A recent study in *S. pneumoniae* strain D39 described that CpsB is essential for parental levels of capsule production during growth under reduced-oxygen conditions, such as met during infection, but not under high-oxygen conditions, such as in the nasopharynx [81]. This activity was independent of its phosphatase activity, suggesting that CpsB participates in alternative capsule regulatory systems. Anyhow, whether the increased *cps2D* expression levels (*cps2B* in *S. pneumoniae*), found during *S. suis* infection in our study affects capsule production remains to be evaluated. Overall, the expression of virulence factors was highly variable at different infection sites and this may be related to different host defense mechanisms or acquisition of nutrients.

### Identification of genes that contribute to survival of S. suis during infection

We next hypothesized that genes with a differential expression during infection of pigs might contribute to the survival of *S. suis* within the host. We selected two genes coding for proteins belonging to different functional categories, one encoding the substrate-binding component of a methionine transporter (SSU1577, *metQ*) and the other encoding an NADH oxidase (SSU0682, *nox*). These genes were up- and downregulated in one infection site as compared to THB growth (Figure 3(c,d)), respectively. Additionally, we included SSU0288, the highest downregulated gene at all sites of infection as compared to THB growth (Figure 3(d)). To understand the contribution of these proteins to infection, we generated deletion mutants of the three selected genes in strain 10. Strain 10 is the reference virulent strain for *S. suis* serotype 2 and our model strain for coinfection experiments [71]. We opted to use coinfection experiments since such experiments allow comparing the growth of the wild type and a coinoculated isogenic mutant derivative within the same animal [71]. Since the wild type is included as an internal control, interindividual variations are drastically reduced, providing a precise and sensitive method of establishing the fitness of a mutant. Coinfections were performed intravenously, and the CFU number of each strain was determined at multiple infection sites to monitor bacterial dissemination.

**Figure 4.**
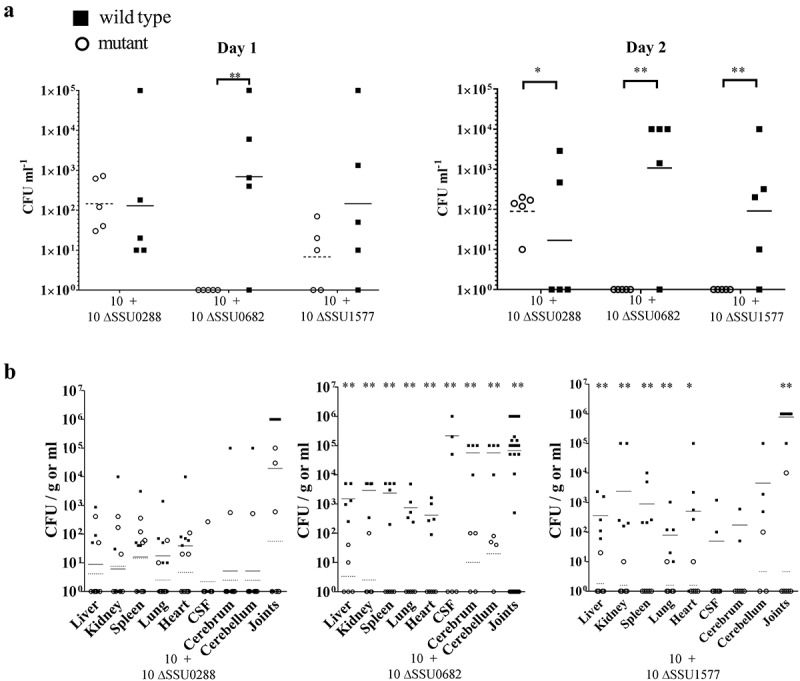
Bacterial load in organs after intravenous infection of pigs with wild-type and mutant strains. (a) Bacterial loads of wild type and mutant derivatives in blood 1 and 2 days postinfection (dpi). (b) Bacterial loads in different organs at four dpi is shown. Each symbol represents one individual animal and geometric means are indicated by horizontal lines for wild type (continuous) or mutants (discontinuous). Before statistical analysis, data were log_10_ converted and unpaired t-test was used for statistical comparison. Statistically significant differences were analyzed with GraphPad Prism 6 and marked at a *p* of < 0.05 (one asterisk) or *p* < 0.001 (two asterisks).

**Figure 5.**
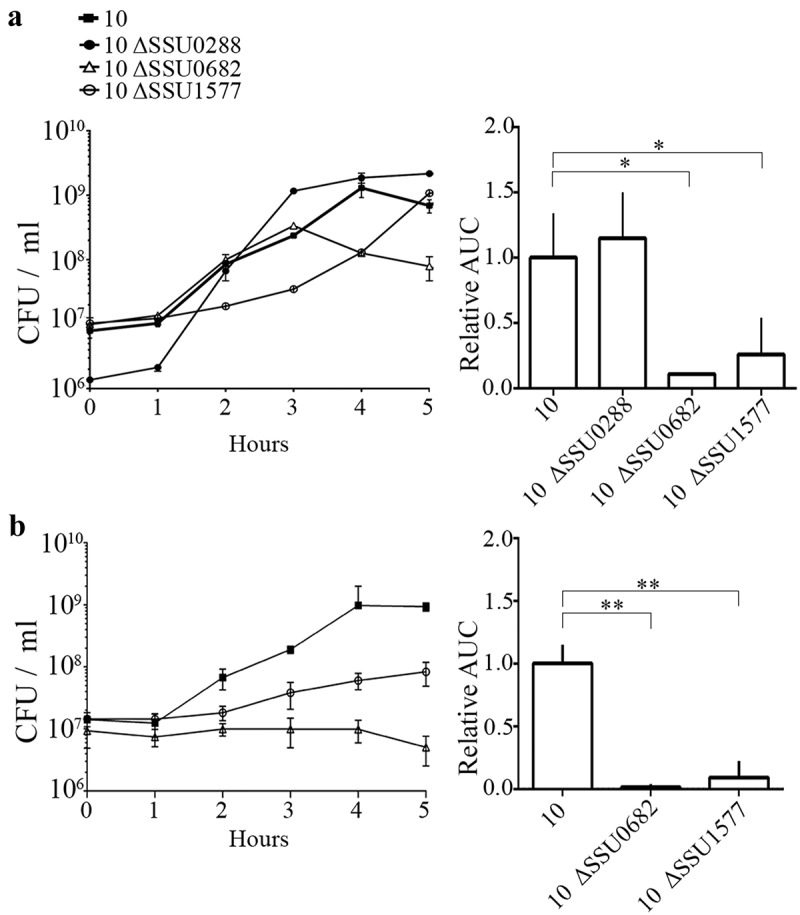
Growth of S. suis strain 10 and mutant derivatives in (a) porcine serum and (b) porcine whole blood. Bacterial strains were grown in THB medium, diluted, and then incubated in porcine serum or fresh porcine blood. The numbers of live bacteria were determined by CFU counting at different time points as indicated. Experiments were repeated at least three times. For both panels, the left graft shows the growth of one representative experiment with three technical replicates. The right graft shows the area under the curve (AUC) relative to the wild-type strain, arbitrarily set to 1, of at least three biological replicates with average and standard deviation. Statistically significant differences were analyzed with GraphPad Prism 6 (unpaired statistical t-test) and are indicated with one asterisk (*p* < 0.05) or two asterisks (*p* < 0.01).

*S. suis* strains 10 ΔSSU0682 and 10 ΔSSU1577 were attenuated for growth in the blood of infected pigs, as evidenced by a reduction in CFU of streptomycin-resistant mutants compared to those of the wild type in blood samples recovered 1 and 2 days postinfection (Figure 4(a)). In contrast, mutant 10 ΔSSU0288 was isolated from blood in similar amounts as the wild type after 1 day, but showed a higher survival than the wild type after 2 days. Remarkably, the interindividual variation of bacteria isolated of the ΔSSU0288 mutant from blood was lower than of the wild type. Compared to the parent, 10 ΔSSU0682 and 10 ΔSSU1577 were hardly isolated from organs (Figure 4(b)), while 10 ΔSSU0288 was isolated in similar amounts as the wild type (Figure 4(b)). To confirm that the attenuation of the ΔSSU0682 and ΔSSU1577 mutants for growth in blood, all mutants were first incubated in porcine serum *in vitro*; under these conditions, erythrocytes and leukocytes are absent. Bacteria were recovered at various time points to assess the number of live bacteria. As expected, the mutants ΔSSU0682 and ΔSSU1577 revealed an altered growth in porcine serum compared to the parent (Figure 5(a)). The mutant ΔSSU0682 had a similar growth rate as the wild type, but rapidly lyses at the end of the log phase. In contrast, the mutant ΔSSU1577 had an extended lag phase, but once in the log phase, the growth rate does not seem to be much different from the wild type. Then, strains 10 ΔSSU0682 and 10 ΔSSU1577 were grown in fresh porcine blood. In contrast to serum, the results revealed a severe attenuation of both mutants as compared to the parent (Figure 5(b)), possibly as consequence of the activity of host cells (e.g. bacterial killing by leukocytes). Together, these data confirmed that the production of MetQ and NADH oxidase is relevant for bacteremia and, thus, for dissemination to the organs, while reduction in the production of the putative exporter encoded by SSU0288 is apparently important to adapt to stress conditions. To the best of our knowledge, the role of this exporter during the infection has not been studied before. MetQ is a surface-exposed lipoprotein and part of an ABC transporter involved in the acquisition of methionine in *S. pneumoniae* [82]. The MetQ protein of *S. suis* shares >70% of sequence identity with the *S. pneumoniae* homologues. The growth of a *metQ* mutant of *S. pneumoniae* was also impaired in blood, but, in contrast to *S. suis*, the mutant was not attenuated for systemic infection, with exception of reduced bacterial loads in spleen [82]. Besides, mixed infection models showed no evidence of attenuation of the mutant in the colonization of the nasopharynx [82]. Apparently, the role of MetQ in infection is different between both species. Several works support a relevant role of NADH oxidase during infection. A *nox* mutant of *S. suis* exhibited impaired growth under oxidative stress conditions [83]. When the mutant was created in the Chinese *S. suis* serotype 2 strain SC19, virulence was attenuated in mouse and pig models [84], in line with our data in strain 10. In *S. pneumoniae*, NADH oxidase was detected in cell-wall fractions suggesting that the protein might also be cell surface-exposed, although it lacks a signal sequence for secretion. Deletion of *nox* in *S. pneumoniae* reduced significantly the adhesion to epithelial cells and the mutant was attenuated in virulence and colonization in diverse mice models [84,85]. Interestingly, mice immunized with recombinant NADH oxidase survived a lethal challenge with *S. pneumoniae* [83]. The NADH oxidase reduces free oxygen to H_2_O_2_ or H_2_O through electron transfer from NADH resulting in the production of NAD^+^. During glycolysis, bacteria produce NADH from NAD^+^. This enzyme balances the NAD^+^/NADH ratio and thus glycolysis can proceed. NADH oxidase is important coping oxidative stress conditions generated by innate immune responses, i.e. reactive oxygen species produced by host phagocytes, to evade the host immune system, in agreement with our whole blood assays. Yet, why *nox* expression is downregulated in pericardia remains to be elucidated. Together with previous findings, this study shows that both proteins, MetQ and NADH oxidase, are relevant factors for *S. suis* infection and that they are, therefore, attractive candidates for the design of vaccines, e.g. live attenuated vaccines.

### Concluding remarks

Analysis of the bacterial transcriptome during infection provided valuable insights into the adaptation of *S. suis* to infection of the porcine host. Specific gene expression profiles were identified in brain, joint, and heart tissues or blood. Although many of the differentially expressed genes encode proteins previously proposed to be relevant for infection of the host, many others were identified here for the first time including regulators, potential transporters, and integral membrane and secreted proteins without assigned functions. It will be important to investigate the role of these novel structures in future works. Our work highlights that the pathogen undergoes a rapid adaptive response that remodels bacterial metabolism and virulence (Figure 3(b–e)), probably governed in part by a repertoire of differentially expressed transcriptional regulators (Figure 3(a)). Clearly, different structures are differently required at specific niches in the process of dissemination and expansion of the disease. We also show that NADH oxidase and MetQ play a relevant role in the survival of the pathogen in blood (Figures 4 and 5), a prerequisite for colonization of organs. In contrast, downregulation of the putative ion exporter encoded by SSU0288 is beneficial for the adaptation of *S. suis* to blood (Figures 4 and 5).

This study focused on transcriptomic profiles of bacteria isolated from blood and tissue-associated bacteria during infection. Future studies focused on transcriptomic profiles of different niche locations within organs or during adhesion and invasion, a prerequisite for streptococcal infection, or following the dynamics of infection, will complete the gene expression profiles of this pathogen during host interaction.
