# Supplementary material for: In vivo transcriptomes of Streptococcus suis reveal genes required for niche-specific adaptation and pathogenesis
Source: Virulence. 2019 Apr 7;10(1):334–51. doi: 10.1080/21505594.2019.1599669 (PMC6527017; doi:10.1080/21505594.2019.1599669)
Supplement: Supplemental Material [file kvir-10-01-1599669-s001.zip › Figure S1_JAB.pdf]

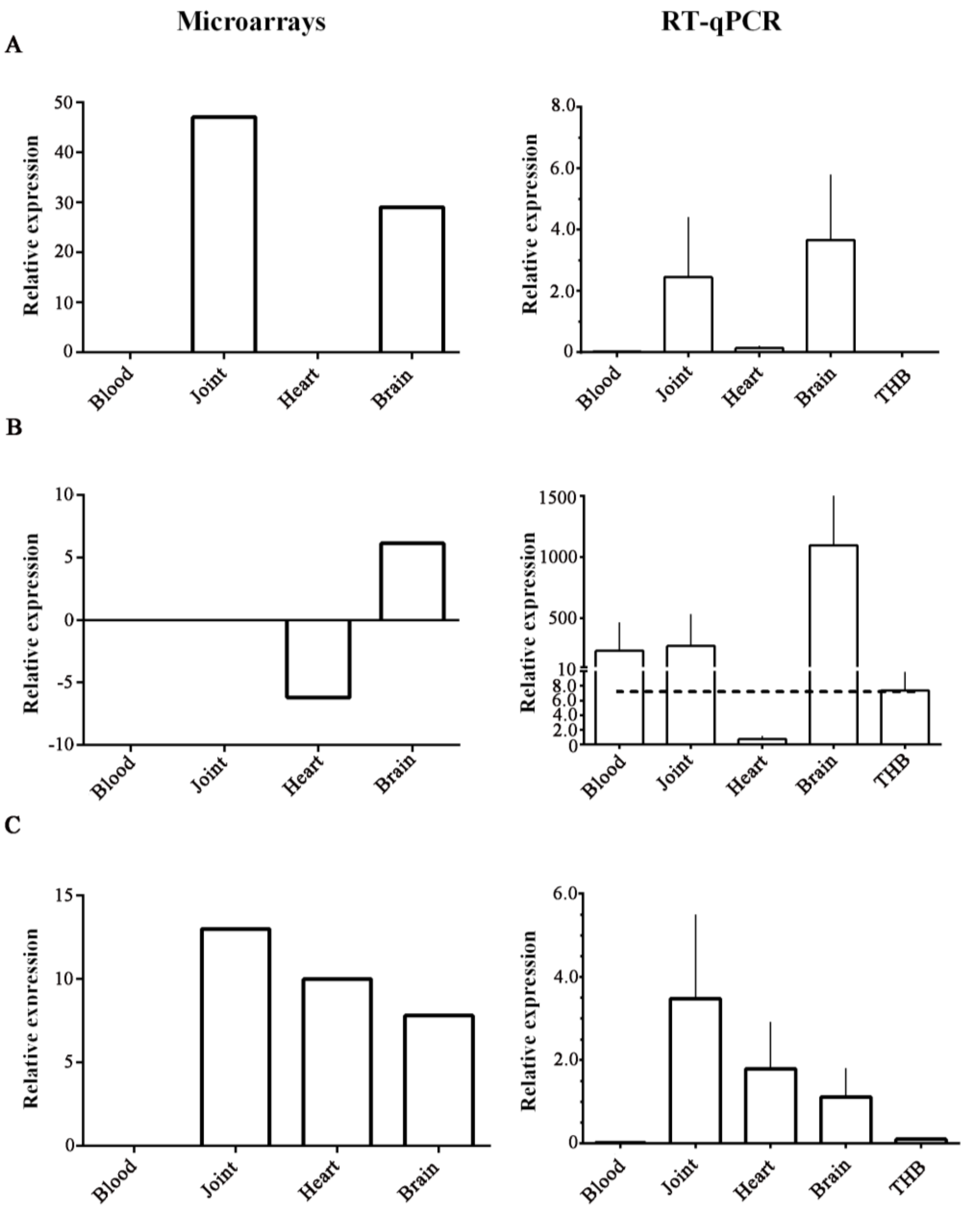

Figure S1 Comparison of expression profiles of (A) SSU0357 (B) SSU0899 and (C) SSU1849 obtained by microarrays (left panels) and RT-qPCR (right panels) of bacteria recovered from different sites of infection (blood, meningeal fluid [brain], joint fluid [joint], and pericardial fluid [heart]) and from THB cultures. The number and origin of the samples used is depicted in Table S2. (PDF).
