# Supplementary material for: In vivo transcriptomes of Streptococcus suis reveal genes required for niche-specific adaptation and pathogenesis
Source: Virulence. 2019 Apr 7;10(1):334–51. doi: 10.1080/21505594.2019.1599669 (PMC6527017; doi:10.1080/21505594.2019.1599669)
Supplement: Supplemental Material [file kvir-10-01-1599669-s001.zip › Table S1 and S2_JAB.docx]

**Table S1.** Primers used in this study.

| Target Gene^a^ | Putative function | Primer | Sequence (5’- 3’) |
| --- | --- | --- | --- |
| RT-qPCR |  |  |  |
| SSU0135 | Putative folate transporter (*orf2*) | Orf2-fw | CTACGGCTGGTTCTTCTATCGAA |
|  |  | Orf2-rev | GCAATCGGTGTCATGATAAAGG |
| SSU1849 | Amylopullulanase (*apuA*) | 1849_Fw | ACTTCATGATGACAGACCGTTTCTAT |
|  |  | 1849_Rev | AAGTAGTCCAGCTTGGCTGTCACT |
| SSU0357 | glucose/maltose-specific transporter | 0357_Fw | AGCACCAATTCTTGCACCTTTT |
|  |  | 0357_Rev | ATGTCATAGGTGCCACCAAGTG |
| SSU0899 | galactose-6-phosphate isomerase LacA subunit (*lacA)* | 0899_Fw | TTGGCATTGTGATTGATGCTTAT |
|  |  | 0899_Rev | GAATTGTTGTGTCCTCTTGTCATATAGG |
| SSU1753 | Prolyl tRNA synthase (*proS*) | pros_Fw | ACAACCCAACGGTCGAGATC |
|  |  | pros_Rev | CACTCGTGCAGGATTGGAGTT |
| SSU1908 | DNA mismatch repair protein (*mutS*) | muts_Fw | CACGCGCCTCCAACATCT |
|  |  | muts_Rev | CCAATTCCAATCTGGGCAAT |
| SSU1103 | Gyrase A (*gyrA*) | gyra_Fw | GAATCCCGGTTTCCGTTACA |
|  |  | gyra_Rev | AATCGCCAACTCTTCCTATCGTT |
| Detection of *S. suis* | | | |
|  |  |  |  |
| SSU0234 | Glutamate dehydrogenase (*gdh*) | JP4 | GCAGCGTATTCTGTCAAACG |
|  |  | JP5 | CCATGGACAGATAAAGATGG |
|  |  |  |  |
| Preparation of knockouts | | | |
|  | | | |
|  | | | |
| SSU0288 Putative metal exporter (SSU0288) SSU0288 L rev AAAATCGACCTGCAGTAGTTTGCTCTT  CCTTATTTAGAAT  SSU0288 L for AAGTCCAAATCATAGGGATAGAA  SSU0288 R for TGCATAGGGTACCGATACCGAGGCATGAT  TCTCTTAACCA  SSU0288 R rev ATTTCTCTCATGCGTGGAATTCCC | | | |
| SSU0682 NADH oxidase (*nox*) FSSU0682-L1 ttgtcatgattttctccttatttc  RSSU0682-L2 acgaacgaaaatcgacctgcta  gcaccgacaacaacgattttag  Fssu0682-R2 ttagaaaacaataaacccttg  catggcaatccaagaaggtgta  Rssu0682-R1 aagccgtgtagcatttagc | | | |
| SSU1577 Amino-acid receptor (*metQ*) F1577-L1 tagcgcgtgaattggcagaca  R1577-L2 acgaacgaaaatcgacctgcga  atcgctcaagctcatcacacct  f1577-R2 ttagaaaacaataaacccttgca  tttgggaaaaatcagagca  R1577-R1 taccccgcgaccataga | | | |
| Spectinomycin-resistance cassette  FSPEC gcaggtcgattttcgttcgt  Rspec atgcaagggtttattgttttctaa | | | |
|  | | | |

^a^ Gene loci based on the genome sequence of P1/7 are provided.

**Table S2.** Site of infection of piglets infected with strain S735-pCOM1-*orf2* used for extraction of *S. suis* RNA included in microarray studies and RT-qPCR studies.

| **Piglet** | **Site of infection** |
| --- | --- |
| 1 | Joint |
| 2 | Joint, Heart, Blood, Brain |
| 3 | Brain |
| 4 | Joint, Brain, Blood |
| 5 | Heart, Joint |
| 6 | Joint (2), Blood |
| 7 | Joint, Brain |
| 8 | Brain |
| 9 | Brain |
| 10 | Brain, Heart, Joint |
