# Supplementary material for: In vivo transcriptomes of Streptococcus suis reveal genes required for niche-specific adaptation and pathogenesis
Source: Virulence. 2019 Apr 7;10(1):334–51. doi: 10.1080/21505594.2019.1599669 (PMC6527017; doi:10.1080/21505594.2019.1599669)
Supplement: Supplemental Material [file kvir-10-01-1599669-s001.zip › Table S3_jab.docx]

**Table S3**. Genes differently expressed *in vivo* during experimental infection as determined in microarrays studies.

| **Probe / Gene locus^a^** | **Putative function^b^** | **EEC number^c^** | **COG^d^** | | | | **p value** | **Global fold change (vitro / vivo)^e^** |
| --- | --- | --- | --- | --- | --- | --- | --- | --- |
|  |  |  | **1** | **2** | **3** | **4** |  |  |
| SSU0288 | putative cation-transporting ATPase |  | P |  |  |  | <0.0001 | 33.58 |
| SSU1740 | 3-isopropylmalate dehydrogenase | 1.1.1.85 | C | E |  |  | 0.0003 | 17.21 |
| SSU1553 | Putative membrane protein |  |  |  |  |  | <0.0001 | 14.8 |
| 891591_GMORF_rev1691 |  |  |  |  |  |  | 0.0001 | 13.28 |
| 8067_GMORF_rev1018 |  |  |  |  |  |  | 0.0030 | 12.23 |
| SSU1310 | transcription antiterminator |  |  |  |  |  | <0.0001 | 11.39 |
| 7917_GMORF_rev1507 | multiple sugar-binding protein precursor |  |  |  |  |  | 0.0007 | 11.33 |
| SSU1309 | Beta-glucoside-specific phospho transferase system (PTS), II-ABC component | 2.7.1.69 |  |  |  |  | <0.0001 | 11.28 |
| 7917_GMORF_rev247 | alpha,alpha-phosphotrehalase/trehalose-6-phosphate hydrolase |  |  |  |  |  | <0.0001 | 11.27 |
| SSU0216 | putative trehalose-6-phosphate hydrolase | 3.2.1.93 | G |  |  |  | 0.0006 | 11.26 |
| SSU1667 | 30S ribosomal protein S15 |  |  |  |  |  | 0.0006 | 11.06 |
| 7917_GMORF684 | integral membrane protein |  |  |  |  |  | 0.0001 | 11.00 |
| 7917_GMORF_rev1891 | 2-isopropylmalate synthase |  |  |  |  |  | 0.0008 | 10.37 |
| SSU1555 | putative membrane protein |  |  |  |  |  | <0.0001 | 10.24 |
| SSU1171 | binding-protein-dependent transport system membrane protein |  |  |  |  |  | <0.0001 | 10.23 |
| SSU0298 | zinc-binding dehydrogenase |  | C | R |  |  | 0.0005 | 9.42 |
| SSU1308 | 6-phospho-beta-glucosidase | 3.2.1.86 |  |  |  |  | <0.0001 | 9.24 |
| 8067_GMORF1617 |  |  |  |  |  |  | <0.0001 | 8.96 |
| 8067_GMORF_rev1204 |  |  |  |  |  |  | <0.0001 | 8.95 |
| SSU1552 | putative membrane protein |  |  |  |  |  | <0.0001 | 8.89 |
| SSU0978 | putative membrane protein |  |  |  |  |  | <0.0001 | 8.88 |
| SSU1277 | hypothetical protein |  |  |  |  |  | 0.0041 | 8.79 |
| SSU1280 | putative membrane protein |  | R |  |  |  | <0.0001 | 8.62 |
| SSU1554 | putative membrane protein |  |  |  |  |  | <0.0001 | 8.47 |
| 8067_GMORF_rev2012 |  |  |  |  |  |  | 0.0019 | 8.45 |
| SSU1556 | putative membrane protein |  |  |  |  |  | <0.0001 | 8.01 |
| 8067_GMORF_rev223 |  |  |  |  |  |  | 0.0008 | 7.92 |
| 7917_GMORF1491 |  |  |  |  |  |  | 0.0044 | 7.79 |
| SSU1741 | 2-isopropylmalate synthase | 2.3.3.13 | E |  |  |  | 0.0024 | 7.71 |
| 8067_GMORF_rev1647 |  |  |  |  |  |  | 0.0001 | 7.55 |
| 7917_GMORF_rev1890 |  |  |  |  |  |  | 0.0006 | 7.46 |
| 7917_GMORF1609 |  |  |  |  |  |  | 0.0009 | 7.30 |
| 8067_GMORF1614 |  |  |  |  |  |  | <0.0001 | 7.21 |
| SSU0878 | acetyltransferase family protein |  |  |  |  |  | 0.0040 | 6.90 |
| SSU0481 | putative exported protein |  |  |  |  |  | 0.0038 | 6.90 |
| SSU1833 | hypothetical protein |  |  |  |  |  | 0.0032 | 6.87 |
| SSU0242 | putative membrane protein |  | S |  |  |  | 0.0002 | 6.79 |
| 7917_GMORF345 |  |  |  |  |  |  | 0.0007 | 6.77 |
| 8067_GMORF_rev2059 |  |  |  |  |  |  | 0.0009 | 6.72 |
| SSU1711 | ROK family protein |  |  |  |  |  | 0.0032 | 6.65 |
| SSU0669 | hypothetical protein |  |  |  |  |  | 0.0037 | 6.63 |
| 7917_GMORF286 |  |  |  |  |  |  | 0.0001 | 6.55 |
| 8067_GMORF133 | YhgE/Pip C-terminal domain protein |  |  |  |  |  | 0.0006 | 6.54 |
| SSU0964 | conserved hypothetical protein |  |  |  |  |  | <0.0001 | 6.53 |
| 8067_GMORF_rev1853 |  |  |  |  |  |  | <0.0001 | 6.19 |
| SSU0480 | RNA polymerase sigma factor protein |  | K |  |  |  | 0.0019 | 6.18 |
| 7917_GMORF1097 |  |  |  |  |  |  | 0.0085 | 5.97 |
| 7917_GMORF1607 |  |  |  |  |  |  | 0.0017 | 5.97 |
| SSU0630 | putative membrane protein (pseudogene) |  |  |  |  |  | <0.0001 | 5.95 |
| 8067_GMORF_rev1641 |  |  |  |  |  |  | 0.0004 | 5.94 |
| SSU0873 | glycogen synthase | 2.4.1.21 | G |  |  |  | 0.0030 | 5.90 |
| SSU0217 | sugar phosphotransferase system (PTS), IIABC |  | G |  |  |  | 0.0056 | 5.86 |
| SSU1708 | binding-protein-dependent transport system membrane protein |  |  |  |  |  | 0.0046 | 5.84 |
| SSU1279 | LemA family protein |  | S |  |  |  | 0.0025 | 5.79 |
| 891591_GMORF_rev1689 |  |  |  |  |  |  | 0.0081 | 5.68 |
| SSU1738 | 3-isopropylmalate dehydratase small subunit | 4.2.1.33 | E |  |  |  | 0.0051 | 5.64 |
| SSU0870 | glucose-1-phosphate adenylyltransferase | 2.7.7.27 | G |  |  |  | 0.0003 | 5.63 |
| 8067_GMORF690 |  |  |  |  |  |  | 0.0054 | 5.61 |
| SSU1170 | extracellular solute-binding protein |  |  |  |  |  | 0.0082 | 5.61 |
| SSU0278 | heat-inducible transcription repressor HrcA |  | K |  |  |  | <0.0001 | 5.59 |
| SSU1425 | SprT-like protein |  | S |  |  |  | 0.0043 | 5.55 |
| SSU1551 | putative DNA-binding protein |  | K |  |  |  | 0.0008 | 5.53 |
| 891591_GMORF354 |  |  |  |  |  |  | 0.0038 | 5.38 |
| SSU1671 | putative ABC transporter, ATP-binding protein |  | O |  |  |  | <0.0001 | 5.34 |
| SSU0302 | putative transcription regulation protein |  |  |  |  |  | 0.0027 | 5.33 |
| 891591_GMORF_rev1079 |  |  |  |  |  |  | 0.0041 | 5.26 |
| SSU0450 | putative signal peptidase I 3 | 3.4.21.89 |  |  |  |  | 0.0053 | 5.26 |
| SSU0871 | glucose-1-phosphate adenylyltransferase pseudogene | 2.7.7.27 |  |  |  |  | 0.0002 | 5.25 |
| SSU1087 | putative phospho-2-dehydro-3-deoxyheptonate aldolase 2 | 2.5.1.54 | E |  |  |  | 0.0032 | 5.21 |
| 8067_GMORF_rev2060 |  |  |  |  |  |  | 0.0014 | 5.21 |
| 7917_GMORF1608 |  |  |  |  |  |  | 0.0006 | 5.13 |
| 891591_GMORF_rev1644 |  |  |  |  |  |  | 0.0018 | 5.08 |
| SSU1686 | dihydroxy-acid dehydratase | 4.2.1.9 | E | G |  |  | 0.0010 | 5.06 |
| SSU0411 | conserved hypothetical protein |  |  |  |  |  | <0.0001 | 5.05 |
| SSU1500 | Dps-like peroxide resistance protein Dpr |  | P |  |  |  | 0.0013 | 5.04 |
| 7917_GMORF45 |  |  |  |  |  |  | 0.0005 | 4.94 |
| 8067_GMORF373 |  |  |  |  |  |  | 0.0094 | 4.88 |
| SSU0977 | predicted membrane protein |  | S |  |  |  | <0.0001 | 4.87 |
| 7917_GMORF_rev1947 |  |  |  |  |  |  | 0.0006 | 4.83 |
| 891591_GMORF_rev1253 |  |  |  |  |  |  | 0.0003 | 4.82 |
| SSU1669 | putative cysteine desulfurase | 4.4.1.16 | E |  |  |  | <0.0001 | 4.82 |
| 7917_GMORF_rev1508 |  |  |  |  |  |  | <0.0001 | 4.81 |
| SSU0187 | Xaa-Pro dipeptidyl-peptidase |  | R |  |  |  | 0.0023 | 4.63 |
| 7917_GMORF_rev1742 |  |  |  |  |  |  | 0.0007 | 4.60 |
| SSU1201 | putative surface-anchored protein |  |  |  |  |  | 0.0022 | 4.49 |
| 8067_GMORF_rev554 |  |  |  |  |  |  | 0.0053 | 4.46 |
| SSU1670 | conserved hypothetical protein |  | O |  |  |  | <0.0001 | 4.42 |
| SSU1371 | multiple sugar-binding transport system permease protein |  |  |  |  |  | 0.0068 | 4.38 |
| SSU1840 | conserved hypothetical protein |  | R |  |  |  | 0.0023 | 4.33 |
| SSU1790 |  |  |  |  |  |  | 0.0047 | 4.31 |
| SSU1498 | ABC-2 type transporter membrane protein |  | V |  |  |  | <0.0001 | 4.24 |
| 8067_GMORF309 |  |  |  |  |  |  | <0.0001 | 4.17 |
| 8067_GMORF_rev986 |  |  |  |  |  |  | 0.0021 | 4.15 |
| SSU0244 | putative membrane protein |  | S |  |  |  | 0.0025 | 4.09 |
| 7917_GMORF1098 |  |  |  |  |  |  | 0.0022 | 4.05 |
| SSU0903 | putative DNA-binding protein |  |  |  |  |  | <0.0001 | 4.03 |
| 7917_GMORF_rev1827 |  |  |  |  |  |  | 0.0072 | 3.95 |
| 8067_GMORF_rev1699 |  |  |  |  |  |  | <0.0001 | 3.95 |
| SSU0299 | hydrolase, alpha/beta fold family |  | R |  |  |  | <0.0001 | 3.94 |
| SSU1619 | putative sucrose-6-phosphate hydrolase | 3.2.1.26 | G |  |  |  | 0.0023 | 3.90 |
| SSU0724 | putative membrane protein |  |  |  |  |  | 0.0001 | 3.90 |
| 8067_GMORF1665 |  |  |  |  |  |  | 0.0004 | 3.86 |
| SSU1499 | ABC transporter ATP-binding protein |  | V |  |  |  | 0.0005 | 3.85 |
| SSU0388 | response regulator protein |  | T | K |  |  | <0.0001 | 3.83 |
| 8067_GMORF_rev1852 |  |  |  |  |  |  | 0.0002 | 3.81 |
| SSU0963 | conserved hypothetical protein (pseudogene) |  |  |  |  |  | 0.0070 | 3.79 |
| SSU0413 | putative membrane protein |  |  |  |  |  | <0.0001 | 3.78 |
| SSU0130 | putative membrane protein |  |  |  |  |  | 0.0008 | 3.78 |
| SSU1418 | prolipoprotein diacylglyceryl transferase | 2.4.99. | M |  |  |  | <0.0001 | 3.65 |
| SSU1391 | putative membrane protein |  |  |  |  |  | <0.0001 | 3.63 |
| SSU1668 | NifU-like protein |  | C |  |  |  | <0.0001 | 3.56 |
| SSU0874 | 1,4-alpha-glucan branching enzyme | 2.4.1.18 | G |  |  |  | 0.0011 | 3.55 |
| SSU1465 | putative geranyltranstransferase | 2.5.1.10 |  |  |  |  | <0.0001 | 3.52 |
| SSU1600 | (3R)-hydroxymyristoyl-[acyl carrier protein] dehydratase | 4.2.1. | I |  |  |  | 0.0044 | 3.51 |
| SSU0147 | 60 kDa chaperonin |  | O |  |  |  | <0.0001 | 3.49 |
| SSU0839 | putative esterase |  | S |  |  |  | 0.0098 | 3.48 |
| SSU1088 | shikimate 5-dehydrogenase | 1.1.1.25 | E |  |  |  | <0.0001 | 3.47 |
| SSU0001 | chromosomal replication initiator protein |  | L |  |  |  | <0.0001 | 3.47 |
| 7917_GMORF1842 |  |  |  |  |  |  | 0.0003 | 3.47 |
| SSU0972 | haloacid dehalogenase-like hydrolase |  | R |  |  |  | <0.0001 | 3.38 |
| SSU1214 | copper-transporting ATPase | 3.6.3.4 | P |  |  |  | 0.0001 | 3.38 |
| SSU1709 | binding-protein-dependent transport system membrane protein |  |  |  |  |  | 0.0091 | 3.37 |
| SSU0976 | PadR family regulatory protein |  | K |  |  |  | 0.0024 | 3.36 |
| SSU0691 | cobalt transport family protein |  |  |  |  |  | 0.0087 | 3.35 |
| SSU0841 | conserved hypothetical protein |  |  |  |  |  | 0.0008 | 3.33 |
| 8067_GMORF_rev2294 |  |  |  |  |  |  | <0.0001 | 3.31 |
| 8067_GMORF263 |  |  |  |  |  |  | 0.0013 | 3.29 |
| SSU0651 | type I restriction-modification system restriction protein |  |  |  |  |  | <0.0001 | 3.28 |
| 7917_GMORF_rev1941 |  |  |  |  |  |  | 0.0004 | 3.27 |
| 8067_GMORF259 |  |  |  |  |  |  | <0.0001 | 3.25 |
| 8067_GMORF545 |  |  |  |  |  |  | <0.0001 | 3.25 |
| 891591_GMORF616 |  |  |  |  |  |  | 0.0002 | 3.24 |
| SSU0236 | putative membrane protein |  | M |  |  |  | 0.0002 | 3.23 |
| SSU0261 | aldehyde-alcohol dehydrogenase 2 | 1.2.1.10 | C |  |  |  | 0.0014 | 3.22 |
| SSU1369 | sucrose phosphorylase | 2.4.1.7 |  |  |  |  | 0.0043 | 3.21 |
| SSU1287 | putative exported protein |  |  |  |  |  | <0.0001 | 3.18 |
| SSU1086 | putative phospho-2-dehydro-3-deoxyheptonate aldolase 1 | 2.5.1.54 | E |  |  |  | <0.0001 | 3.16 |
| SSU0301 | NADH: flavin oxidoreductase / NADH oxidase family protein |  |  |  |  |  | 0.0006 | 3.16 |
| SSU0969 | conserved hypothetical protein |  | S |  |  |  | 0.0037 | 3.10 |
| SSU1589 | type I restriction-modification system S protein |  |  |  |  |  | 0.0007 | 3.09 |
| SSU1829 | rhomboid family membrane protein |  | R |  |  |  | 0.0001 | 3.09 |
| 7917_GMORF56 |  |  |  |  |  |  | 0.0089 | 3.08 |
| 7917_GMORF_rev1826 |  |  |  |  |  |  | 0.0010 | 3.05 |
| SSU0386 | putative membrane protein |  | S |  |  |  | <0.0001 | 3.05 |
| SSU1573 | conserved hypothetical protein |  | S |  |  |  | 0.0014 | 3.05 |
| 8067_GMORF_rev1713 |  |  |  |  |  |  | 0.0027 | 3.05 |
| SSU1212 | conserved hypothetical protein |  |  |  |  |  | 0.0081 | 3.04 |
| SSU0951 | putative phosphate ABC transporter permease protein |  | P |  |  |  | <0.0001 | 3.04 |
| 891591_GMORF_rev1930 |  |  |  |  |  |  | 0.0006 | 3.03 |
| SSU0254 | putative surface-anchored protein (pseudogene)" |  |  |  |  |  | 0.0062 | 3.02 |
| SSU0254 | putative surface-anchored protein (pseudogene)" |  |  |  |  |  | 0.0062 | 3.02 |
| SSU0970 | putative membrane protein |  |  |  |  |  | <0.0001 | 3.01 |
| SSU0758 | carbohydrate kinase |  | G |  |  |  | 0.0084 | 2.99 |
| SSU1666 | conserved hypothetical protein |  | O |  |  |  | 0.0015 | 2.97 |
| SSU1274 | type I restriction-modification system R protein |  |  |  |  |  | <0.0001 | 2.97 |
| SSU1023 | putative permease |  | R |  |  |  | <0.0001 | 2.94 |
| SSU1382 | putative membrane protein |  |  |  |  |  | <0.0001 | 2.93 |
| SSU1681 | acetolactate synthase small subunit | 2.2.1.6 | E |  |  |  | 0.0001 | 2.88 |
| SSU1947 | putative tRNA (5-methylaminomethyl-2-thiouridylate)-methyltransferase | 2.1.1.61 | J |  |  |  | 0.0051 | 2.87 |
| 8067_GMORF_rev1763 |  |  |  |  |  |  | <0.0001 | 2.87 |
| SSU0410 | glyoxalase/bleomycin resistance protein/dioxygenase superfamily protein |  | E |  |  |  | 0.0003 | 2.78 |
| SSU0245 | PadR-like family regulatory protein |  | K |  |  |  | 0.0054 | 2.75 |
| SSU0888 | putative membrane protein |  |  |  |  |  | 0.0007 | 2.75 |
| SSU0387 | sensor histidine kinase |  | T |  |  |  | <0.0001 | 2.75 |
| 891591_GMORF_rev1404 |  |  |  |  |  |  | 0.0012 | 2.74 |
| SSU0145 | putative membrane protein |  |  |  |  |  | 0.0056 | 2.72 |
| SSU1520 | aminopeptidase C | 3.4.22.40 | E |  |  |  | 0.0069 | 2.71 |
| SSU1515 | putative phosphodiesterase |  | R |  |  |  | 0.0005 | 2.71 |
| SSU1590 | type I restriction-modification system R | 3.1.21.3 |  |  |  |  | 0.0035 | 2.70 |
| SSU0279 | GrpE protein (HSP-70 cofactor) |  | O |  |  |  | 0.0025 | 2.70 |
| SSU0152 | putative endopeptidase |  | O |  |  |  | 0.0013 | 2.69 |
| SSU1024 | putative membrane protein |  | S |  |  |  | 0.0004 | 2.69 |
| 891591_GMORF163 |  |  |  |  |  |  | 0.0046 | 2.68 |
| 7917_GMORF_rev1409 |  |  |  |  |  |  | <0.0001 | 2.67 |
| SSU1452 | acetyl-CoA acetyltransferase | 2.3.1.9 | I |  |  |  | 0.0003 | 2.67 |
| SSU1406 | putative bacitracin export permease protein |  |  |  |  |  | 0.0003 | 2.65 |
| 6388v2_GMORF_rev482 |  |  |  |  |  |  | 0.0054 | 2.65 |
| 891591_GMORF_rev1216 |  |  |  |  |  |  | 0.0026 | 2.64 |
| 7917_GMORF327 |  |  |  |  |  |  | 0.0003 | 2.63 |
| SSU1830 | 5-formyltetrahydrofolate cyclo-ligase family protein |  | H |  |  |  | 0.0008 | 2.61 |
| SSU1599 | biotin carboxylase subunit of acetyl-CoA carboxylase | 6.3.4.14 | I |  |  |  | 0.0098 | 2.61 |
| 8067_GMORF_rev158 |  |  |  |  |  |  | 0.0015 | 2.61 |
| SSU0211 | conserved hypothetical protein |  | L |  |  |  | 0.0009 | 2.60 |
| SSU1651 | HIT-family protein |  | F | G | R |  | <0.0001 | 2.60 |
| 8067_GMORF128 |  |  |  |  |  |  | 0.0011 | 2.58 |
| SSU0640 | type III restriction-modification system modification enzyme (fragment) |  |  |  |  |  | 0.0017 | 2.56 |
| 7917_GMORF555 |  |  |  |  |  |  | 0.0009 | 2.56 |
| SSU1278 | flotillin family protein |  | S |  |  |  | 0.0010 | 2.54 |
| SSU0185 | putative tagatose-6-phosphate aldose/ketose isomerase |  | M |  |  |  | 0.0081 | 2.54 |
| SSU1068 | CAAX amino terminal protease family membrane protein |  |  |  |  |  | 0.0035 | 2.53 |
| 891591_GMORF_rev2047 |  |  |  |  |  |  | 0.0004 | 2.52 |
| SSU1453 | 3-hydroxy-3-methylglutaryl coenzyme A synthase | 2.3.3.10 | I |  |  |  | <0.0001 | 2.49 |
| 6388v2_GMORF1003 |  |  |  |  |  |  | 0.0081 | 2.46 |
| SSU1299 | putative membrane protein |  |  |  |  |  | 0.0023 | 2.45 |
| 8067_GMORF129 |  |  |  |  |  |  | 0.0053 | 2.45 |
| SSU1082 | putative oligopeptidase | 3.4.24. | E |  |  |  | <0.0001 | 2.42 |
| SSU1370 | multiple sugar-binding transport system permease protein |  |  |  |  |  | <0.0001 | 2.40 |
| SSU1784 | putative thiamin pyrophosphokinase | 2.7.6.2 | H |  |  |  | 0.0002 | 2.39 |
| SSU1019 | ATP synthase A chain | 3.6.3.14 | C |  |  |  | 0.0002 | 2.38 |
| SSU1682 | acetolactate synthase large subunit | 2.2.1.6 | E | H |  |  | 0.0008 | 2.38 |
| SSU1645 | putative N utilization substance protein A |  | K |  |  |  | 0.0006 | 2.37 |
| SSU0746 | putative polysaccharide deacetylase |  | G |  |  |  | 0.0008 | 2.36 |
| 891591_GMORF_rev1643 |  |  |  |  |  |  | 0.0003 | 2.36 |
| 891591_GMORF614 |  |  |  |  |  |  | 0.0001 | 2.33 |
| 7917_GMORF_rev654 |  |  |  |  |  |  | 0.0082 | 2.32 |
| SSU0304 | putative lipase |  |  |  |  |  | 0.0001 | 2.31 |
| SSU1517 | glutamate racemase | 5.1.1.3 | M |  |  |  | <0.0001 | 2.30 |
| SSU1074 | putative membrane protein |  |  |  |  |  | 0.0084 | 2.29 |
| SSU1413 | ABC-type glycine betaine transport system protein |  | M |  |  |  | 0.0031 | 2.25 |
| 8067_GMORF_rev2186 |  |  |  |  |  |  | 0.0001 | 2.24 |
| SSU1454 | 3-hydroxy-3-methylglutaryl-coenzyme A reductase | 1.1.1.88 | I |  |  |  | 0.0075 | 2.24 |
| SSU1783 | RmuC family protein |  | S |  |  |  | 0.0081 | 2.23 |
| SSU1871 | putative membrane protein |  |  |  |  |  | 0.0010 | 2.23 |
| SSU1140 | putative membrane protein |  |  |  |  |  | 0.0069 | 2.23 |
| 7917_GMORF_rev2026 |  |  |  |  |  |  | 0.0019 | 2.22 |
| SSU1795 | toxin-antitoxin system, toxin protein |  | S |  |  |  | 0.0016 | 2.21 |
| SU1152A |  |  |  |  |  |  | 0.0012 | 2.20 |
| SSU1222 | multi-drug resistance efflux pump |  | G |  |  |  | <0.0001 | 2.14 |
| SSU0971 | conserved hypothetical protein |  |  |  |  |  | <0.0001 | 2.14 |
| SSU1224 | Formamido pyrimidine-DNA glycosylase | 3.2.2.23 | L |  |  |  | 0.0030 | 2.13 |
| SSU1180 | putative cation-transporting ATPase |  | P |  |  |  | 0.0036 | 2.11 |
| SSU1047 | LacI family regulatory protein |  |  |  |  |  | 0.0006 | 2.07 |
| 8067_GMORF2127 |  |  |  |  |  |  | 0.0098 | 2.07 |
| SSU0437 | putative membrane protein |  |  |  |  |  | 0.0003 | 2.05 |
| SSU1017 | putative ATP synthase delta chain | 3.6.3.14 | C |  |  |  | 0.0027 | 2.05 |
| SSU0418 | putative DNA-binding protein |  |  |  |  |  | 0.0065 | 2.04 |
| SSU0652 | type I restriction-modification system modification protein (pseudogene) |  |  |  |  |  | 0.0040 | 2.03 |
| SSU1417 | putative exported protein |  | R |  |  |  | 0.0012 | 2.02 |
| 8067_GMORF_rev1163 |  |  |  |  |  |  | 0.0005 | 2.01 |
| 891591_GMORF_rev1035 |  |  |  |  |  |  | 0.0022 | 2.01 |
| SSU1396 | putative ADP-ribose pyrophosphatase | 3.6.1.13 |  |  |  |  | 0.0003 | 1.99 |
| 8067_GMORF_rev256 |  |  |  |  |  |  | 0.0005 | 1.99 |
| SSU1679 | threonine dehydratase biosynthetic | 4.3.1.19 | E |  |  |  | 0.0048 | 1.98 |
| 891591_GMORF_rev511 |  |  |  |  |  |  | 0.0094 | 1.98 |
| SSU1531 | putative helicase |  | K | L |  |  | 0.0025 | 1.97 |
| SSU1226 | diacylglycerol kinase | 2.7.1.107 | M |  |  |  | <0.0001 | 1.96 |
| SSU0002 | DNA polymerase III, beta chain | 2.7.7.7 | L |  |  |  | 0.0021 | 1.96 |
| SSU1127 | putative N-acetylmuramoyl-L-alanine amidase |  | M |  |  |  | 0.0001 | 1.94 |
| 891591_GMORF702 |  |  |  |  |  |  | 0.0020 | 1.93 |
| SSU0508 | glutamate 5-kinase | 2.7.2.11 | E |  |  |  | 0.0004 | 1.93 |
| SU1152B |  |  |  |  |  |  | 0.0023 | 1.92 |
| SSU1512 | segregation and condensation protein A |  | S |  |  |  | 0.0003 | 1.92 |
| SSU0058 | Holliday junction DNA helicase RuvA |  | L |  |  |  | 0.0038 | 1.91 |
| SSU1210 | ABC transporter permease protein |  | M |  |  |  | 0.0059 | 1.89 |
| SSU1081 | putative membrane protein |  |  |  |  |  | <0.0001 | 1.88 |
| 8067_GMORF_rev1500 |  |  |  |  |  |  | 0.0085 | 1.87 |
| 8067_GMORF_rev1467 |  |  |  |  |  |  | 0.0084 | 1.85 |
| SSU1255 | RNA polymerase sigma factor RpoD |  | K |  |  |  | 0.0041 | 1.85 |
| SSU0881 | hypothetical protein |  |  |  |  |  | 0.0029 | 1.84 |
| SU1872A |  |  |  |  |  |  | 0.0030 | 1.84 |
| SSU0280 | chaperone protein DnaK (heat shock protein 70) |  | O |  |  |  | 0.0085 | 1.84 |
| SSU0973 | peptidase T | 3.4.11.14 | E |  |  |  | 0.0001 | 1.83 |
| SSU1827 | glycerol-3-phosphate dehydrogenase [NAD(P)+] | 1.1.1.94 | C |  |  |  | 0.0002 | 1.83 |
| 8067_GMORF509 |  |  |  |  |  |  | 0.0094 | 1.83 |
| SSU1503 | putative membrane protein |  | S |  |  |  | 0.0079 | 1.82 |
| 891591_GMORF361 |  |  |  |  |  |  | 0.0033 | 1.82 |
| SSU1768 | conserved hypothetical protein |  |  |  |  |  | 0.0027 | 1.81 |
| SSU1678 | putative membrane protein |  | O |  |  |  | 0.0028 | 1.80 |
| SSU1626 | CorA-like Mg2+ transporter protein |  | P |  |  |  | 0.0021 | 1.79 |
| SSU1794 | conserved hypothetical protein |  |  |  |  |  | 0.0016 | 1.76 |
| SSU1680 | ketol-acid reductoisomerase | 1.1.1.86 | E | H |  |  | 0.0084 | 1.73 |
| SSU0057 | DNA mismatch repair protein MutL |  | L |  |  |  | 0.0078 | 1.73 |
| SSU0052 | hypothetical protein |  |  |  |  |  | 0.0081 | 1.72 |
| SSU1290 | putative phosphoglucosamine mutase |  | G |  |  |  | 0.0003 | 1.71 |
| SSU0417 | conserved hypothetical protein |  |  |  |  |  | <0.0001 | 1.70 |
| 8067_GMORF457 |  |  |  |  |  |  | 0.0007 | 1.67 |
| SSU0576 | conserved hypothetical protein |  | S |  |  |  | 0.0041 | 1.67 |
| SSU1030 | putative haloacid dehalogenase-like hydrolase |  | R |  |  |  | 0.0091 | 1.67 |
| SSU1208 | DNA polymerase I | 2.7.7.7 | L |  |  |  | 0.0018 | 1.66 |
| 8067_GMORF_rev1460 |  |  |  |  |  |  | 0.0005 | 1.65 |
| SSU0430 | UDP-N-acetylmuramoylalanine--D-glutamate ligase | 6.3.2.9 | M |  |  |  | 0.0066 | 1.65 |
| 8067_GMORF434 |  |  |  |  |  |  | 0.0085 | 1.64 |
| SSU1189 | metallo-beta-lactamase superfamily protein |  | R |  |  |  | 0.0066 | 1.64 |
| SSU0444 | putative ATP-dependent Clp protease ATP-binding subunit |  | O |  |  |  | 0.0001 | 1.63 |
| SSU0475 | glycosyl hydrolases family protein |  | M |  |  |  | 0.0050 | 1.63 |
| SSU1511 | segregation and condensation protein B |  | K |  |  |  | 0.0017 | 1.63 |
| SSU0014 | hypoxanthine-guanine phosphoribosyltransferase | 2.4.2.8 | F |  |  |  | 0.0055 | 1.61 |
| SSU0656 | putative lipoprotein |  |  |  |  |  | 0.0032 | 1.61 |
| 8067_GMORF2337 |  |  |  |  |  |  | 0.0083 | 1.61 |
| SSU1190 | sensor histidine kinase |  | T |  |  |  | 0.0001 | 1.60 |
| SSU1123 | putative glycosyltransferase |  | M |  |  |  | 0.0053 | 1.60 |
| SSU0748 | homoserine kinase | 2.7.1.39 | E |  |  |  | 0.0069 | 1.59 |
| 8067_GMORF_rev1164 |  |  |  |  |  |  | 0.0027 | 1.59 |
| SSU0497 | FAD dependent oxidoreductase |  |  |  |  |  | 0.0004 | 1.58 |
| SSU1292 | putative membrane protein |  | S |  |  |  | 0.0026 | 1.57 |
| 7917_GMORF902 |  |  |  |  |  |  | 0.0021 | 1.57 |
| SSU1467 | putative exodeoxyribonuclease VII large subunit | 3.1.11.6 | L |  |  |  | 0.0015 | 1.54 |
| SSU1796 | toxin-antitoxin system, antitoxin protein |  | D |  |  |  | 0.0057 | 1.52 |
| 7917_GMORF495 |  |  |  |  |  |  | 0.0062 | 1.52 |
| SSU1080 | hypothetical protein |  |  |  |  |  | <0.0001 | 1.51 |
| SSU1038 | phosphoenolpyruvate-protein phosphotransferase | 2.7.3.9 | G |  |  |  | 0.0066 | 1.51 |
| 891591_GMORF_rev2027 |  |  |  |  |  |  | 0.0096 | 1.51 |
| SSU0213 | ribonuclease HIII | 3.1.26.4 | L |  |  |  | 0.0070 | 1.50 |
| SSU1302 | metallo-beta-lactamase superfamily protein |  | R |  |  |  | 0.0008 | 1.50 |
| SSU1122 | putative polysaccharide export ABC transporter permease protein |  | G | M |  |  | 0.0071 | 1.49 |
| SSU1544 | putative membrane protein |  |  |  |  |  | 0.0019 | 1.45 |
| SSU1025 | glucose-6-phosphate 1-dehydrogenase | 1.1.1.49 | G |  |  |  | 0.0082 | 1.45 |
| SSU0015 | putative cell division protease FtsH |  | O |  |  |  | 0.0012 | 1.45 |
| SSU1314 | putative membrane protein |  | R |  |  |  | 0.0069 | 1.41 |
| SSU0561 | putative RNA methyltransferase |  | J |  |  |  | 0.0033 | 1.41 |
| SSU0564 | putative NADPH-dependent FMN reductase |  | R |  |  |  | 0.0018 | 1.39 |
| SSU1130 | putative dTDP-glucose-4,6-dehydratase | 4.2.1.46 | M |  |  |  | 0.0044 | 1.38 |
| SSU0370 | putative penicillin-binding protein 1A |  | M |  |  |  | 0.0098 | 1.34 |
| SSU1545 | putative helicase |  | L | K | J |  | 0.0096 | 1.33 |
| SSU0326 | glutamyl-tRNA amidotransferase subunit B | 6.3.5. | J |  |  |  | 0.0068 | -1.21 |
| SSU1320 | enolase | 4.2.1.11 | G |  |  |  | 0.0053 | -1.32 |
| SSU1150 | Major Facilitator Superfamily protein |  | P |  |  |  | 0.0010 | -1.33 |
| SSU0434 | cell division protein FtsZ |  | D |  |  |  | 0.0092 | -1.34 |
| SSU0521 | putative glycosyl transferase |  |  |  |  |  | 0.0017 | -1.35 |
| SSU0341 | isochorismatase family protein (pseudogene) |  |  |  |  |  | 0.0032 | -1.35 |
| SSU0231 | mechanosensitive ion channel protein |  | M |  |  |  | 0.0087 | -1.36 |
| SSU1108 | putative membrane protein |  |  |  |  |  | 0.0014 | -1.38 |
| SSU0780 | putative exported protein |  |  |  |  |  | 0.0061 | -1.38 |
| SSU0470 | lysyl-tRNA synthetase | 6.1.1.6 | J |  |  |  | 0.0071 | -1.38 |
| SSU0479 | putative phosphoenolpyruvate carboxylase | 4.1.1.31 | C |  |  |  | 0.0021 | -1.39 |
| SSU1139 | adenine phosphoribosyltransferase | 2.4.2.7 | F |  |  |  | 0.0099 | -1.40 |
| SSU1538 | ATP-binding protein |  | K |  |  |  | 0.0008 | -1.40 |
| SSU1955 | putative membrane protein |  | S |  |  |  | 0.0049 | -1.41 |
| SSU0420 | aspartate--ammonia ligase | 6.3.1.1 | E |  |  |  | 0.0010 | -1.43 |
| SSU1034 | ArsR family regulatory protein |  | K |  |  |  | 0.0027 | -1.44 |
| SSU1923 | aspartyl-tRNA synthetase | 6.1.1.12 | J |  |  |  | 0.0069 | -1.45 |
| SSU1767 | transcriptional regulator |  |  |  |  |  | 0.0090 | -1.45 |
| SSU0516 | putative chain length determinant protein |  | M |  |  |  | 0.0058 | -1.46 |
| SSU1530 | conserved hypothetical protein |  |  |  |  |  | 0.0029 | -1.46 |
| SSU1897 | conserved hypothetical protein |  |  |  |  |  | 0.0022 | -1.46 |
| SSU0945 | response regulator protein |  | T | K |  |  | 0.0094 | -1.46 |
| SSU0319 | putative aminotransferase |  | E |  |  |  | 0.0046 | -1.48 |
| SSU0563 | UDP-galactopyranose mutase | 5.4.99.9 | M |  |  |  | 0.0007 | -1.50 |
| SSU1688 | conserved hypothetical protein |  |  |  |  |  | 0.0042 | -1.50 |
| SSU0334 | haloacid dehalogenase-like hydrolase |  | R |  |  |  | 0.0098 | -1.50 |
| SSU0783 | nucleoside diphosphate kinase | 2.7.4.6 | F |  |  |  | 0.0006 | -1.51 |
| SSU1489 | conserved hypothetical protein |  | S |  |  |  | <0.0001 | -1.51 |
| 891591_GMORF_rev985 |  |  |  |  |  |  | 0.0072 | -1.53 |
| SSU1434 | putative flavoprotein |  |  |  |  |  | 0.0077 | -1.54 |
| SSU1758 | adenylosuccinate synthetase | 6.3.4.4 | F |  |  |  | 0.0032 | -1.56 |
| SSU1743 | aminopeptidase PepS | 3.4.11.-" | E |  |  |  | 0.0006 | -1.57 |
| SSU0523 | putative membrane protein |  |  |  |  |  | <0.0001 | -1.57 |
| SSU1960 | inosine-5'-monophosphate dehydrogenase | 1.1.1.205 | F |  |  |  | 0.0001 | -1.58 |
| SSU1326 | septation ring formation regulator |  | D |  |  |  | 0.0020 | -1.58 |
| SSU1010 | putative competence associated protein |  |  |  |  |  | 0.0001 | -1.58 |
| SSU0782 | putative GTP-binding protein |  | R |  |  |  | <0.0001 | -1.61 |
| 891591_GMORF_rev1892 |  |  |  |  |  |  | 0.0055 | -1.62 |
| 891591_GMORF_rev1432 |  |  |  |  |  |  | 0.0051 | -1.62 |
| SSU0730 | LysR family regulatory protein |  | K |  |  |  | 0.0043 | -1.63 |
| SSU1011 | UDP-N-acetylglucosamine 1-carboxyvinyltransferase 1 | 2.5.1.7 | M |  |  |  | <0.0001 | -1.64 |
| SSU1634 | dihydrolipoamide dehydrogenase | 1.8.1.4 | C |  |  |  | 0.0073 | -1.66 |
| SSU1952 | ABC transporter ATP-binding protein |  | P |  |  |  | 0.0003 | -1.66 |
| 891591_GMORF_rev2171 |  |  |  |  |  |  | 0.0046 | -1.66 |
| SSU0858 | conserved hypothetical protein |  | R |  |  |  | 0.0089 | -1.67 |
| SSU0750 | UDP-N-acetylenolpyruvoylglucosamine reductase | 1.1.1.158 | M |  |  |  | 0.0006 | -1.67 |
| SSU0439 | putative cell-division protein DivIVA |  | D |  |  |  | 0.0001 | -1.68 |
| SSU1653 | LytR family regulatory protein |  | K |  |  |  | 0.0002 | -1.68 |
| SSU0628 | putative membrane protein |  |  |  |  |  | 0.0022 | -1.68 |
| SSU0049 | putative DNA-binding protein |  |  |  |  |  | 0.0016 | -1.69 |
| SSU1205 | putative membrane protein |  | S |  |  |  | 0.0046 | -1.69 |
| SSU1779 | RNA pseudouridylate synthase |  | J |  |  |  | 0.0094 | -1.69 |
| SSU0448 | FolD bifunctional protein | 1.5.1.5 | H |  |  |  | 0.0030 | -1.69 |
| 8067_GMORF2128 |  |  |  |  |  |  | 0.0029 | -1.70 |
| SSU0139 | putative exported protein |  |  |  |  |  | 0.0018 | -1.70 |
| SSU1721 | polyribonucleotide nucleotidyltransferase | 2.7.7.8 | J |  |  |  | 0.0001 | -1.70 |
| SSU1550 | S-adenosyl-methyltransferase MraW | 2.1.1.-" | M |  |  |  | 0.0059 | -1.70 |
| SSU1819 | conserved hypothetical protein |  | S |  |  |  | 0.0014 | -1.71 |
| SSU0380 | putative primosomal protein N' |  | L |  |  |  | 0.0011 | -1.74 |
| SSU1806 | ribonuclease P protein component | 3.1.26.5 | J |  |  |  | 0.0016 | -1.74 |
| SSU0797 | putative NADP-dependent L-serine/L-allo-threonine dehydrogenase |  | R |  |  |  | 0.0048 | -1.76 |
| 891591_GMORF_rev1275 |  |  |  |  |  |  | <0.0001 | -1.77 |
| SSU0310 | putative DNA-directed RNA polymerase, delta subunit | 2.7.7.6 | K |  |  |  | <0.0001 | -1.77 |
| SSU0465 | putative exported protein |  | M |  |  |  | 0.0001 | -1.77 |
| 891591_GMORF221 |  |  |  |  |  |  | 0.0088 | -1.78 |
| SSU0096 | 50S ribosomal protein L36 |  | J |  |  |  | 0.0010 | -1.78 |
| SSU0197 | hypothetical protein |  |  |  |  |  | 0.0004 | -1.79 |
| SSU0779 | dihydrofolate reductase | 1.5.1.3 | H |  |  |  | <0.0001 | -1.79 |
| SSU0379 | DNA-directed RNA polymerase omega chain | 2.7.7.6 | K |  |  |  | <0.0001 | -1.79 |
| 891591_GMORF185 |  |  |  |  |  |  | 0.0003 | -1.80 |
| SSU1656 | conserved hypothetical protein |  | R |  |  |  | 0.0002 | -1.80 |
| SSU1629 | single strand binding protein (SSB) |  | L |  |  |  | 0.0036 | -1.81 |
| SSU0921 | conserved hypothetical protein |  |  |  |  |  | 0.0014 | -1.81 |
| SSU1534 | GTP-binding protein EngA |  | R |  |  |  | <0.0001 | -1.81 |
| 891591_GMORF_rev1709 |  |  |  |  |  |  | 0.0007 | -1.81 |
| 7917_GMORF849 |  |  |  |  |  |  | 0.0017 | -1.81 |
| 891591_GMORF874 |  |  |  |  |  |  | 0.0005 | -1.82 |
| 7917_GMORF_rev1856 |  |  |  |  |  |  | 0.0088 | -1.82 |
| SSU0079 | 30S ribosomal protein S17 |  | J |  |  |  | 0.0024 | -1.83 |
| SSU0235 | putative dihydroorotate dehydrogenase | 1.3.3.1 | F |  |  |  | 0.0002 | -1.83 |
| SSU1430 | ribonuclease BN-like family protein |  | S |  |  |  | <0.0001 | -1.83 |
| 891591_GMORF_rev1265 |  |  |  |  |  |  | 0.0001 | -1.84 |
| SSU1689 | hypothetical protein (pseudogene) |  |  |  |  |  | <0.0001 | -1.84 |
| SSU1431 | putative metallopeptidase |  | J |  |  |  | 0.0004 | -1.84 |
| SSU0155 | putative membrane protein |  |  |  |  |  | 0.0066 | -1.85 |
| SSU0728 | 50S ribosomal protein L21 |  | J |  |  |  | 0.0005 | -1.85 |
| 891591_GMORF2140 |  |  |  |  |  |  | 0.0065 | -1.87 |
| SSU0859 | Septum formation protein Maf |  | D |  |  |  | 0.0050 | -1.87 |
| SSU1800 | hypothetical protein |  |  |  |  |  | 0.0001 | -1.87 |
| SSU1441 | DNA polymerase III subunit gamma/tau | 2.7.7.7 | L |  |  |  | <0.0001 | -1.88 |
| SSU1358 | putative exported protein |  |  |  |  |  | <0.0001 | -1.89 |
| 8067_GMORF_rev1900 |  |  |  |  |  |  | 0.0079 | -1.89 |
| SSU0013 | PP-loop family protein |  | D |  |  |  | 0.0029 | -1.89 |
| SSU1791 | putative membrane protein |  | M |  |  |  | 0.0005 | -1.89 |
| SSU0778 | thymidylate synthase | 2.1.1.45 | F |  |  |  | 0.0003 | -1.90 |
| SSU1762 | putative tRNA-dihydrouridine synthase |  | J |  |  |  | 0.0081 | -1.90 |
| SSU0268 | multi antimicrobial extrusion (MATE) family transporter |  | V |  |  |  | 0.0062 | -1.91 |
| SSU0648 | formate--tetrahydrofolate ligase 1 | 6.3.4.3 | F |  |  |  | <0.0001 | -1.91 |
| 7917_GMORF173 |  |  |  |  |  |  | 0.0006 | -1.92 |
| SSU1144 | short chain dehydrogenase |  | R |  |  |  | 0.0008 | -1.92 |
| SSU0769 | conserved hypothetical protein |  | S |  |  |  | <0.0001 | -1.93 |
| 8067_GMORF633 |  |  |  |  |  |  | 0.0006 | -1.93 |
| 8067_GMORF_rev905 |  |  |  |  |  |  | 0.0049 | -1.93 |
| 8067_GMORF_rev1895 |  |  |  |  |  |  | 0.0008 | -1.93 |
| SSU1540 | response regulator protein |  | T | K |  |  | 0.0052 | -1.93 |
| 891591_GMORF1519 |  |  |  |  |  |  | 0.0043 | -1.93 |
| 891591_GMORF_rev1158 |  |  |  |  |  |  | 0.0044 | -1.93 |
| 891591_GMORF_rev1350 |  |  |  |  |  |  | 0.0006 | -1.93 |
| 7917_GMORF168 |  |  |  |  |  |  | 0.0029 | -1.94 |
| 891591_GMORF639 |  |  |  |  |  |  | 0.0063 | -1.95 |
| SSU1009 | putative competence associated endonuclease |  |  |  |  |  | <0.0001 | -1.96 |
| 8067_GMORF778 |  |  |  |  |  |  | 0.0065 | -1.97 |
| SSU1078 | foldase protein PrsA precursor | 5.2.1.8 |  |  |  |  | <0.0001 | -1.97 |
| 891591_GMORF_rev1780 |  |  |  |  |  |  | 0.0026 | -1.97 |
| SSU1673 | adapter protein MecA |  | O | T | N |  | 0.0003 | -1.98 |
| SSU0177 | putative DNA-binding protein |  |  |  |  |  | <0.0001 | -2.00 |
| SSU0153 | glyceraldehyde-3-phosphate dehydrogenase | 1.2.1.12 | G |  |  |  | <0.0001 | -2.01 |
| SSU1920 | putative alpha amylase | 3.2.1.41 | G |  |  |  | 0.0005 | -2.01 |
| SSU1804 | putative ssDNA-binding protein |  | R |  |  |  | <0.0001 | -2.04 |
| SSU1284 | sodium:dicarboxylate symporter family protein |  | E |  |  |  | 0.0045 | -2.04 |
| SSU0095 | translation initiation factor IF-1 |  | J |  |  |  | <0.0001 | -2.04 |
| 8067_GMORF741 |  |  |  |  |  |  | 0.0019 | -2.04 |
| 8067_GMORF_rev2185 |  |  |  |  |  |  | 0.0052 | -2.05 |
| SSU0320 | GTP-sensing transcriptional pleiotropic repressor |  | K |  |  |  | 0.0001 | -2.07 |
| 8067_GMORF345 |  |  |  |  |  |  | 0.0035 | -2.10 |
| SSU0232 | putative lipoprotein |  |  |  |  |  | <0.0001 | -2.10 |
| 8067_GMORF_rev2190 |  |  |  |  |  |  | <0.0001 | -2.11 |
| 891591_GMORF_rev1263 |  |  |  |  |  |  | 0.0003 | -2.12 |
| SSU1254 | conserved hypothetical protein |  | R |  |  |  | 0.0020 | -2.13 |
| SSU1838 | putative deaminase |  |  |  |  |  | 0.0002 | -2.13 |
| SSU0528 | putative membrane protein |  |  |  |  |  | 0.0012 | -2.13 |
| SSU0460 | conserved hypothetical protein |  |  |  |  |  | 0.0006 | -2.15 |
| 8067_GMORF64 |  |  |  |  |  |  | <0.0001 | -2.15 |
| 8067_GMORF546 |  |  |  |  |  |  | 0.0007 | -2.15 |
| 7917_GMORF1913 |  |  |  |  |  |  | 0.0036 | -2.15 |
| SSU0932 | ABC transporter ATP-binding protein |  | R |  |  |  | 0.0001 | -2.16 |
| SSU0827 | sensor histidine kinase |  | T |  |  |  | 0.0005 | -2.16 |
| 891591_GMORF576 |  |  |  |  |  |  | <0.0001 | -2.17 |
| 891591_GMORF779 |  |  |  |  |  |  | 0.0037 | -2.17 |
| SSU0189 | putative beta-lactamase (pseudogene) |  |  |  |  |  | <0.0001 | -2.17 |
| 8067_GMORF_rev2041 |  |  |  |  |  |  | 0.0001 | -2.17 |
| SSU1780 | putative pur operon repressor |  | F |  |  |  | 0.0059 | -2.17 |
| SSU0649 | putative membrane protein |  | S |  |  |  | 0.0017 | -2.20 |
| 891591_GMORF_rev910 |  |  |  |  |  |  | 0.0006 | -2.20 |
| SSU1188 | ABC transporter ATP-binding protein |  |  |  |  |  | 0.0019 | -2.22 |
| SSU1655 | putative P-loop hydrolase |  | R |  |  |  | <0.0001 | -2.22 |
| SSU1921 | putative NrdI-like protein |  | F |  |  |  | 0.0042 | -2.22 |
| SSU1481 | putative transport protein |  | R |  |  |  | <0.0001 | -2.23 |
| SSU0267 | ABC transporter ATP-binding protein |  | R |  |  |  | 0.0072 | -2.24 |
| 891591_GMORF_rev1056 |  |  |  |  |  |  | 0.0020 | -2.24 |
| SSU1757 | putative preprotein translocase subunit |  | U |  |  |  | 0.0042 | -2.24 |
| SSU1736 | polypeptide deformylase | 3.5.1.88 | J |  |  |  | <0.0001 | -2.25 |
| SSU1416 | putative exported protein |  |  |  |  |  | 0.0003 | -2.26 |
| 891591_GMORF_rev562 |  |  |  |  |  |  | 0.0001 | -2.26 |
| SSU0722 | putative L-threonine aldolase |  | E |  |  |  | 0.0053 | -2.27 |
| 8067_GMORF_rev1190 |  |  |  |  |  |  | 0.0012 | -2.27 |
| 7917_GMORF_rev1974 |  |  |  |  |  |  | 0.0001 | -2.27 |
| SSU1488 | radical SAM superfamily protein |  | R |  |  |  | <0.0001 | -2.27 |
| SSU0760 | GMP reductase | 1.7.1.7 | F |  |  |  | 0.0006 | -2.27 |
| SSU1443 | putative membrane protein |  | S |  |  |  | 0.0044 | -2.27 |
| 891591_GMORF572 |  |  |  |  |  |  | 0.0048 | -2.29 |
| SSU0975 | putative transporter protein |  | R |  |  |  | 0.0045 | -2.29 |
| 8067_GMORF_rev856 |  |  |  |  |  |  | 0.0024 | -2.30 |
| SSU0486 | putative membrane protein |  |  |  |  |  | 0.0001 | -2.30 |
| 8067_GMORF231 |  |  |  |  |  |  | 0.0037 | -2.30 |
| SSU1483 | putative rRNA methylase |  | H |  |  |  | <0.0001 | -2.31 |
| SSU0314 | ATP-dependent DNA helicase | 3.6.1. | L | K |  |  | 0.0006 | -2.31 |
| SSU0378 | guanylate kinase | 2.7.4.8 | F |  |  |  | <0.0001 | -2.32 |
| 8067_GMORF_rev2136 |  |  |  |  |  |  | 0.0033 | -2.32 |
| SSU1231 | suilysin (hemolysin) |  |  |  |  |  | <0.0001 | -2.33 |
| SSU0408 | conserved hypothetical protein |  |  |  |  |  | <0.0001 | -2.33 |
| SSU0360 | conserved hypothetical protein |  | S |  |  |  | 0.0006 | -2.34 |
| SSU1328 | DJ-1/PfpI family protein |  | R |  |  |  | <0.0001 | -2.34 |
| SSU0845 | 50S ribosomal protein L7/L12 |  | J |  |  |  | 0.0084 | -2.34 |
| SSU1729 | uracil permease |  | F |  |  |  | 0.0098 | -2.34 |
| 8067_GMORF_rev120 |  |  |  |  |  |  | 0.0006 | -2.34 |
| SSU1638 | putative membrane protein |  | S |  |  |  | 0.0001 | -2.34 |
| 8067_GMORF_rev1529 |  |  |  |  |  |  | 0.0017 | -2.35 |
| SSU0764 | tRNA (Guanine-N(1)-)-methyltransferase | 2.1.1.31 | J |  |  |  | <0.0001 | -2.36 |
| 7917_GMORF769 |  |  |  |  |  |  | 0.0051 | -2.36 |
| 891591_GMORF_rev945 |  |  |  |  |  |  | 0.0092 | -2.37 |
| SSU0398 | conserved hypothetical protein |  | J |  |  |  | 0.0001 | -2.38 |
| SSU1216 | Tellurite resistance protein TehB family protein |  | P |  |  |  | 0.0085 | -2.38 |
| 7917_GMORF_rev1959 |  |  |  |  |  |  | <0.0001 | -2.39 |
| SSU0223 | putative thioredoxin |  | O |  |  |  | 0.0035 | -2.39 |
| 8067_GMORF446 |  |  |  |  |  |  | 0.0007 | -2.39 |
| 8067_GMORF_rev724 |  |  |  |  |  |  | 0.0012 | -2.39 |
| SSU0018 | rod shape-determining protein MreC |  | M |  |  |  | 0.0010 | -2.40 |
| SSU0817 | GntR family regulatory protein |  | K |  |  |  | 0.0031 | -2.40 |
| 7917_GMORF_rev2027 |  |  |  |  |  |  | 0.0004 | -2.42 |
| 891591_GMORF32 |  |  |  |  |  |  | 0.0035 | -2.42 |
| SSU0134 | acetate kinase | 2.7.2.1 |  |  |  |  | <0.0001 | -2.43 |
| SSU0487 | conserved hypothetical protein |  | S |  |  |  | <0.0001 | -2.43 |
| 8067_GMORF_rev118 |  |  |  |  |  |  | 0.0012 | -2.44 |
| 7917_GMORF_rev2076 |  |  |  |  |  |  | <0.0001 | -2.46 |
| 891591_GMORF634 |  |  |  |  |  |  | 0.0001 | -2.46 |
| SSU0477 | putative lipoprotein |  |  |  |  |  | 0.0008 | -2.49 |
| SSU0633 | CAAX amino terminal protease family protein |  |  |  |  |  | 0.0045 | -2.50 |
| SSU0264 | ABC transporter ATP-binding membrane protein |  | V |  |  |  | 0.0085 | -2.51 |
| 891591_GMORF_rev33 |  |  |  |  |  |  | <0.0001 | -2.52 |
| SSU0306 | trigger factor (peptidyl-prolyl isomerase) | 5.2.1.8 | O |  |  |  | 0.0001 | -2.54 |
| SSU0025 | putative acyl carrier protein |  | I | Q |  |  | 0.0001 | -2.55 |
| 8067_GMORF532 |  |  |  |  |  |  | 0.0006 | -2.55 |
| 7917_GMORF527 |  |  |  |  |  |  | 0.0025 | -2.55 |
| SSU1582 | putative haloacid dehalogenase-like hydrolase |  | R |  |  |  | 0.0046 | -2.56 |
| 7917_GMORF185 |  |  |  |  |  |  | <0.0001 | -2.57 |
| 891591_GMORF_rev1916 |  |  |  |  |  |  | 0.0028 | -2.59 |
| SSU1937 | conserved hypothetical protein |  | S |  |  |  | 0.0001 | -2.59 |
| 891591_GMORF_rev1936 |  |  |  |  |  |  | <0.0001 | -2.59 |
| 8067_GMORF_rev1178 |  |  |  |  |  |  | <0.0001 | -2.61 |
| 8067_GMORF72 |  |  |  |  |  |  | 0.0004 | -2.61 |
| SSU0461 | conserved hypothetical protein |  | S |  |  |  | <0.0001 | -2.64 |
| 7917_GMORF_rev222 |  |  |  |  |  |  | 0.0019 | -2.64 |
| 891591_GMORF140 |  |  |  |  |  |  | 0.0010 | -2.65 |
| 891591_GMORF2193 |  |  |  |  |  |  | 0.0001 | -2.65 |
| SSU0284 | extracellular solute-binding protein |  | E | T |  |  | <0.0001 | -2.67 |
| SSU0157 | putative glutamine synthetase | 6.3.1.2 |  |  |  |  | 0.0003 | -2.68 |
| SSU1876 | putative membrane protein |  |  |  |  |  | 0.0016 | -2.69 |
| 891591_GMORF_rev1875 |  |  |  |  |  |  | <0.0001 | -2.70 |
| 7917_GMORF2054 |  |  |  |  |  |  | 0.0002 | -2.72 |
| SSU0285 | transport system membrane protein |  | E |  |  |  | 0.0008 | -2.75 |
| 7917_GMORF_rev1977 |  |  |  |  |  |  | 0.0068 | -2.75 |
| 8067_GMORF_rev2116 |  |  |  |  |  |  | 0.0049 | -2.76 |
| SSU0462 | BioY family protein |  | R |  |  |  | 0.0010 | -2.77 |
| SSU0136 | folylpolyglutamate synthase | 6.3.2.17 | H |  |  |  | <0.0001 | -2.80 |
| 7917_GMORF1353 |  |  |  |  |  |  | <0.0001 | -2.80 |
| 8067_GMORF157 |  |  |  |  |  |  | 0.0027 | -2.81 |
| SSU0257 | 50S ribosomal protein L33 1 |  | J |  |  |  | 0.0045 | -2.81 |
| SSU0266 | putative transporter protein |  | P |  |  |  | 0.0075 | -2.82 |
| SSU1458 | DNA-binding protein HU |  | L |  |  |  | 0.0015 | -2.82 |
| 7917_GMORF_rev1708 |  |  |  |  |  |  | <0.0001 | -2.83 |
| SSU0467 | putative permease |  | V |  |  |  | <0.0001 | -2.83 |
| 7917_GMORF_rev1628 |  |  |  |  |  |  | <0.0001 | -2.84 |
| 8067_GMORF_rev1101 |  |  |  |  |  |  | <0.0001 | -2.87 |
| 7917_GMORF144 |  |  |  |  |  |  | 0.0003 | -2.88 |
| 7917_GMORF670 | thymidylate kinase |  |  |  |  |  | 0.0002 | -2.90 |
| 8067_GMORF_rev1536 |  |  |  |  |  |  | 0.0056 | -2.90 |
| 891591_GMORF1677 |  |  |  |  |  |  | 0.0007 | -2.90 |
| SSU0518 | protein-tyrosine phosphatase Wzh | 3.1.3.48 | G | M |  |  | <0.0001 | -2.92 |
| SSU1367 | uracil phosphoribosyltransferase | 2.4.2.9 | F |  |  |  | <0.0001 | -2.92 |
| 891591_GMORF_rev984 |  |  |  |  |  |  | 0.0011 | -2.93 |
| SSU0156 | MerR family regulatory protein |  | K |  |  |  | 0.0003 | -2.94 |
| 8067_GMORF507 |  |  |  |  |  |  | 0.0011 | -2.94 |
| 891591_GMORF_rev1820 |  |  |  |  |  |  | 0.0003 | -2.96 |
| SSU0742 | 30S ribosomal protein S16 |  | J |  |  |  | 0.0004 | -2.98 |
| 891591_GMORF1687 |  |  |  |  |  |  | <0.0001 | -2.98 |
| 891591_GMORF_rev1429 |  |  |  |  |  |  | 0.0021 | -3.01 |
| SSU1773 | putative surface-anchored serine protease |  | O |  |  |  | 0.0003 | -3.01 |
| SSU1875 | putative membrane protein |  |  |  |  |  | 0.0092 | -3.02 |
| 7917_GMORF_rev1652 |  |  |  |  |  |  | 0.0044 | -3.02 |
| 7917_GMORF_rev2047 |  |  |  |  |  |  | 0.0044 | -3.06 |
| 8067_GMORF535 |  |  |  |  |  |  | 0.0002 | -3.07 |
| SSU0332 | CutC family protein |  | P |  |  |  | <0.0001 | -3.10 |
| SSU0183 | putative glycerophosphodiester phosphodiesterase | 3.1.4.46 | C |  |  |  | <0.0001 | -3.11 |
| SSU1803 | 50S ribosomal protein L34 |  |  |  |  |  | 0.0009 | -3.12 |
| SSU0313 | 50S ribosomal protein L28 |  | J |  |  |  | 0.0007 | -3.13 |
| 7917_GMORF480 |  |  |  |  |  |  | <0.0001 | -3.15 |
| SSU1917 | putative maltose/maltodextrin ABC transport system permease protein |  |  |  |  |  | 0.0002 | -3.17 |
| 8067_GMORF_rev187 |  |  |  |  |  |  | 0.0004 | -3.18 |
| 891591_GMORF797 |  |  |  |  |  |  | 0.0001 | -3.23 |
| SSU0392 | conserved hypothetical protein |  | S |  |  |  | <0.0001 | -3.23 |
| 891591_GMORF_rev1640 |  |  |  |  |  |  | <0.0001 | -3.26 |
| SSU0263 | ABC transporter ATP-binding membrane protein |  | C | O |  |  | 0.0006 | -3.27 |
| 8067_GMORF_rev2135 |  |  |  |  |  |  | 0.0005 | -3.28 |
| SSU0721 | putative 30S ribosomal protein S1 |  | J |  |  |  | <0.0001 | -3.29 |
| SSU1501 | CsbD-like protein |  | S |  |  |  | 0.0002 | -3.31 |
| 891591_GMORF1670 |  |  |  |  |  |  | 0.0016 | -3.36 |
| 7917_GMORF_rev1521 |  |  |  |  |  |  | 0.0024 | -3.37 |
| SSUSC84_0814 | ABC transporter ATP-binding membrane protein |  |  |  |  |  | 0.0065 | -3.41 |
| SSU1388 | putative membrane protein (fragment) |  |  |  |  |  | 0.0001 | -3.48 |
| SSU0606 | putative ferrichrome-binding protein precursor |  | P |  |  |  | 0.0006 | -3.50 |
| 7917_GMORF189 |  |  |  |  |  |  | 0.0064 | -3.51 |
| 891591_GMORF327 |  |  |  |  |  |  | 0.0002 | -3.57 |
| 7917_GMORF_rev1893 |  |  |  |  |  |  | <0.0001 | -3.57 |
| 7917_GMORF880 |  |  |  |  |  |  | <0.0001 | -3.60 |
| 8067_GMORF_rev1637 |  |  |  |  |  |  | 0.0001 | -3.62 |
| 891591_GMORF_rev1050 |  |  |  |  |  |  | 0.0024 | -3.78 |
| 891591_GMORF_rev2043 |  |  |  |  |  |  | 0.0094 | -3.81 |
| SSU1387 | DEAD box helicase family protein |  | L | K | J |  | 0.0002 | -3.83 |
| 8067_GMORF_rev2117 |  |  |  |  |  |  | 0.0048 | -3.91 |
| 7917_GMORF_rev1129 |  |  |  |  |  |  | 0.0016 | -3.94 |
| 8067_GMORF_rev653 |  |  |  |  |  |  | 0.0039 | -3.95 |
| 8067_GMORF_rev1638 |  |  |  |  |  |  | 0.0001 | -4.04 |
| 8067_GMORF_rev1495 |  |  |  |  |  |  | 0.0064 | -4.07 |
| 891591_GMORF_rev1041 |  |  |  |  |  |  | 0.0044 | -4.07 |
| 8067_GMORF_rev1661 |  |  |  |  |  |  | 0.0020 | -4.08 |
| 7917_GMORF49 |  |  |  |  |  |  | 0.0068 | -4.11 |
| SSU0124 | conserved hypothetical protein |  | S |  |  |  | <0.0001 | -4.16 |
| 7917_GMORF_rev1477 |  |  |  |  |  |  | 0.0098 | -4.18 |
| 8067_GMORF498 |  |  |  |  |  |  | 0.0061 | -4.21 |
| SSU1259 | 30S ribosomal protein S21 |  | J |  |  |  | <0.0001 | -4.22 |
| SSU0230 | alanine symporter family protein |  | E |  |  |  | <0.0001 | -4.25 |
| 7917_GMORF_rev2032 |  |  |  |  |  |  | 0.0033 | -4.26 |
| 8067_GMORF_rev1977 |  |  |  |  |  |  | 0.0001 | -4.36 |
| 8067_GMORF_rev1792 |  |  |  |  |  |  | 0.0001 | -4.39 |
| 7917_GMORF_rev147 |  |  |  |  |  |  | 0.0023 | -4.42 |
| 8067_GMORF_rev1372 |  |  |  |  |  |  | 0.0056 | -4.47 |
| SSU1581 | putative permease |  | R |  |  |  | <0.0001 | -4.50 |
| SSU1042 | aconitate hydratase | 4.2.1.3 | C |  |  |  | 0.0002 | -4.51 |
| 891591_GMORF209 |  |  |  |  |  |  | <0.0001 | -4.56 |
| SSU0353 | putative 4-alpha-glucanotransferase | 2.4.1.25 | G |  |  |  | <0.0001 | -4.67 |
| 8067_GMORF_rev1485 |  |  |  |  |  |  | 0.0081 | -4.73 |
| 8067_GMORF_rev1386 |  |  |  |  |  |  | 0.0094 | -4.76 |
| 7917_GMORF265 |  |  |  |  |  |  | 0.0028 | -4.80 |
| SU0121B |  |  |  |  |  |  | <0.0001 | -4.81 |
| SSU0940 | 30S ribosomal protein S20 |  | J |  |  |  | <0.0001 | -4.82 |
| 7917_GMORF_rev1445 |  |  |  |  |  |  | 0.0057 | -4.83 |
| SSUSC84_0861 | hypothetical protein |  |  |  |  |  | 0.0036 | -4.96 |
| 7917_GMORF_rev1640 |  |  |  |  |  |  | 0.0007 | -4.96 |
| SSUSC84_0850 | salK SSUSC84_0814 |  |  |  |  |  | 0.0017 | -4.97 |
| 8067_GMORF_rev1219 |  |  |  |  |  |  | 0.0057 | -4.98 |
| 8067_GMORF_rev1494 |  |  |  |  |  |  | 0.0031 | -4.99 |
| 7917_GMORF_rev745 |  |  |  |  |  |  | 0.0053 | -5.04 |
| 8067_GMORF536 |  |  |  |  |  |  | 0.0056 | -5.07 |
| 7917_GMORF543 |  |  |  |  |  |  | 0.0043 | -5.22 |
| 8067_GMORF239 |  |  |  |  |  |  | <0.0001 | -5.23 |
| 8067_GMORF_rev1398 |  |  |  |  |  |  | 0.0035 | -5.25 |
| SSU1934 | putative exported protein |  |  |  |  |  | 0.0001 | -5.25 |
| 7917_GMORF_rev1467 |  |  |  |  |  |  | <0.0001 | -5.29 |
| 7917_GMORF_rev1154 |  |  |  |  |  |  | 0.0001 | -5.30 |
| 8067_GMORF_rev1327 |  |  |  |  |  |  | 0.0036 | -5.45 |
| SU0121A |  |  |  |  |  |  | <0.0001 | -5.50 |
| 891591_GMORF_rev1215 |  |  |  |  |  |  | 0.0091 | -5.54 |
| 8067_GMORF433 |  |  |  |  |  |  | <0.0001 | -5.58 |
| 7917_GMORF_rev722 |  |  |  |  |  |  | 0.0069 | -5.61 |
| 8067_GMORF556 |  |  |  |  |  |  | 0.0001 | -5.62 |
| 891591_GMORF_rev1017 |  |  |  |  |  |  | 0.0027 | -5.66 |
| 8067_GMORF_rev1676 |  |  |  |  |  |  | 0.0016 | -5.88 |
| 7917_GMORF_rev1540 |  |  |  |  |  |  | 0.0001 | -5.90 |
| SSU1776 | 50S ribosomal protein L33 |  | J |  |  |  | <0.0001 | -5.94 |
| 7917_GMORF_rev1696 |  |  |  |  |  |  | <0.0001 | -5.98 |
| 7917_GMORF688 |  |  |  |  |  |  | 0.0010 | -6.00 |
| 7917_GMORF261 |  |  |  |  |  |  | 0.0006 | -6.06 |
| 8067_GMORF_rev2113 |  |  |  |  |  |  | 0.0014 | -6.25 |
| 8067_GMORF_rev105 |  |  |  |  |  |  | 0.0014 | -6.83 |
| SSU1808 | argininosuccinate synthase | 6.3.4.5 | E |  |  |  | 0.0001 | -6.87 |
| 8067_GMORF_rev1773 |  |  |  |  |  |  | 0.0003 | -6.87 |
| SSUSC84_0862 | putative methyltransferase |  |  |  |  |  | 0.0007 | -7.03 |
| 7917_GMORF412 |  |  |  |  |  |  | 0.0009 | -7.23 |
| SSU0354 | putative glycogen phosphorylase | 2.4.1.1 | G |  |  |  | <0.0001 | -7.29 |
| 8067_GMORF_rev2071 | Polyribonucleotide nucleotidyltransferase |  |  |  |  |  | 0.0019 | -7.38 |
| 7917_GMORF387 |  |  |  |  |  |  | <0.0001 | -7.42 |
| SSU1344 | putative DNA-binding phage protein |  |  |  |  |  | 0.0007 | -7.44 |
| SSU0720 | conserved hypothetical protein |  |  |  |  |  | 0.0005 | -7.96 |
| 7917_GMORF_rev725 | delta protein / ATPase (Enterococcus/lactococcus) |  |  |  |  |  | 0.0036 | -7.98 |
| SSU1807 | argininosuccinate lyase | 4.3.2.1 | E |  |  |  | 0.0001 | -8.32 |
| 7917_GMORF_rev2091 | membrane protein |  |  |  |  |  | 0.0006 | -8.88 |
| 8067_GMORF_rev1697 | transcriptional regulator, PadR-like family |  |  |  |  |  | 0.0010 | -8.97 |
| 8067_GMORF_rev1396 |  |  |  |  |  |  | 0.0005 | -8.98 |
| SSU1849 | putative surface-anchored amylopullulanase |  | G |  |  |  | <0.0001 | -9.28 |
| 8067_GMORF_rev1376 | Tn5252 transposon protein [Streptococcus agalactiae ATCC 13813] |  |  |  |  |  | 0.0009 | -9.64 |
| SSU0368 | cold shock protein |  |  |  |  |  | <0.0001 | -10.89 |
| 6388v2_GMORF492 |  |  |  |  |  |  | 0.0001 | -11.61 |
| SSU0356 | endonuclease/exonuclease/phosphatase family protein |  | R |  |  |  | <0.0001 | -12.09 |
| 8067_GMORF_rev906 | conserved hypothetical protein [Leishmania mexicana] |  |  |  |  |  | 0.0006 | -13.06 |
| 8067_GMORF380 | glucose-specific phosphotransferase system, IIABC component |  |  |  |  |  | <0.0001 | -15.34 |
| 7917_GMORF_rev748 | hypothetical protein HMPREF0491_02092 [Lachnospiraceae] |  |  |  |  |  | <0.0001 | -15.77 |
| 7917_GMORF50 | ABC-2 type transporter |  |  |  |  |  | <0.0001 | -16.37 |
| 6388v2_GMORF_rev902 |  |  |  |  |  |  | <0.0001 | -17.81 |
| SSU0357 | putative glucose-specific phosphotransferase system, IIABC component | 2.7.1.69 | G |  |  |  | <0.0001 | -18.32 |
| SSU1809 | putative membrane protein |  |  |  |  |  | <0.0001 | -20.98 |

^a^ Gene loci according to genome sequences on which the probes used in the microarrays were based.

^b^ Putative function of gene products as derived from public genome annotations.

^c^ The number of the Ligand Database for Enzyme Nomenclature (KEGG) is specified.

^d^ Functional classification of gene products based on Clusters of Orthologous Groups.

^e^ The global fold change difference in vitro / in vivo was calculated considering data derived from all infection sites using Genespring GX software.
