# Supplementary material for: In vivo transcriptomes of Streptococcus suis reveal genes required for niche-specific adaptation and pathogenesis
Source: Virulence. 2019 Apr 7;10(1):334–51. doi: 10.1080/21505594.2019.1599669 (PMC6527017; doi:10.1080/21505594.2019.1599669)
Supplement: Supplemental Material [file kvir-10-01-1599669-s001.zip › Table S5_JAB.docx]

| **Pathways** | **Blood** | |  | **Heart** | |  | **Brain** | |  | **Joint** | |
| --- | --- | --- | --- | --- | --- | --- | --- | --- | --- | --- | --- |
|  | Down  (186) | Up  (220) |  | Down  (123) | Up  (99) |  | Down  (159) | Up  (102) |  | Down  (54) | Up  (57) |
| General met | 40(21%) | 13(10%) |  | 21(17%) | 15(15%) |  | 29 (18%) | 18(18%) |  | 8(15%) | 11(19%) |
| Biosynthesis of secondary metabolites | 23(12%) | 6(5%) |  | 9(7%) | 4(4%) |  | 13 (7%) | 7(7%) |  | 6(11%) | 6(11%) |
| Biosynthesis of amino acids | 7(4%) | 4(3%) |  | 3(2%) | 3 (3%) |  | 4 (3%) | 5(5%) |  | 3(6%) | 2(4%) |
| Microbial met in diverse environments | 12(6%) | 4(3%) |  | 4(3%) | 4 (4%) |  | 4 (3%) | 9(9%) |  |  | 2(3%) |
| Alanine, aspartate and glutamate met |  | 2(2%) |  |  | 2 (2%) |  |  |  |  |  | 2(4%) |
| Amino sugar and nucleotide sugar met | 4(2%) |  |  | 5(4%) |  |  |  | 2(2%) |  |  |  |
| Arginine biosynthesis |  | 2(2%) |  |  | 2 (2%) |  |  |  |  |  | 2(4%) |
| Ascorbate and aldarate met | 2(1%) |  |  |  |  |  |  |  |  |  |  |
| Biotin met | 3(2%) |  |  |  |  |  | 3(2%) |  |  |  |  |
| Butanoate met | 3(2%) |  |  | 2(2%) |  |  | 4 (3%) |  |  |  |  |
| Carbon met | 5(3%) | 3(2%) |  |  | 3 (3%) |  | 3(2%) | 7(7%) |  |  | 2(4%) |
| C5 branched dibasic acid met | 2(1%) |  |  |  |  |  | 2(1%) |  |  |  |  |
| Chloroalkane and chloroalkene degradation | 2(1%) |  |  |  |  |  |  |  |  |  |  |
| Citrate cycle |  |  |  |  |  |  |  | 2(2%) |  |  |  |
| Cyanoamino acid met | 2(1%) |  |  |  |  |  |  |  |  |  |  |
| D-glutamine and D glutamate met |  |  |  | 2(2%) |  |  |  |  |  |  |  |
| Degradation of aromatic compounds | 2(1%) |  |  |  |  |  |  |  |  |  |  |
| Fatty acid biosynthesis | 7 (4%) |  |  |  |  |  | 10 (6%) |  |  |  |  |
| Fatty acid degradation | 2(1%) |  |  | 2(2%) |  |  |  |  |  |  |  |
| Folate biosynthesis |  | 2(2%) |  |  |  |  | 3(2%) |  |  |  |  |
| Fructose and mannose met | 2(1%) |  |  |  |  |  |  |  |  |  |  |
| Galactose met | 2(1%) |  |  | 4(3%) | 5 (5%) |  |  |  |  |  |  |
| Glycerolipid met | 2(1%) |  |  |  |  |  |  |  |  |  |  |
| Glycine, serine and threonine met |  | 2(2%) |  | 2(2%) |  |  |  | 2(2%) |  |  |  |
| Glycolysis/gluconeogenesis | 5(3%) | 2(2%) |  | 2(2%) |  |  | 2(1%) | 3(3%) |  |  |  |
| Glyoxylate and dicarboxylate met |  |  |  |  | 2 (2%) |  |  | 3(3%) |  |  |  |
| Naphthalene degradation | 2(1%) |  |  |  |  |  |  |  |  |  |  |
| 2-oxocarboxylic acid met | 4(2%) |  |  |  |  |  | 2(1%) |  |  |  |  |
| Oxidative phosphorylation |  | 2(2%) |  |  |  |  | 4(3%) |  |  |  |  |
| One carbon pool by folate |  |  |  |  |  |  |  | 3(3%) |  |  |  |
| Pantothenate and CoA biosynthesis | 4(2%) |  |  |  |  |  | 3(2%) |  |  |  |  |
| Pentose phosphate pathway | 4(2%) |  |  |  |  |  |  |  |  |  |  |
| Pentose and glucuronate interconversions | 4(2%) |  |  | 2(2%) |  |  |  |  |  |  |  |
| Phenylalanine, tyrosine and tryptophan biosynthesis | 2(1%) |  |  |  |  |  |  |  |  | 2(4%) |  |
| PTS | 5(3%) |  |  | 2(2%) |  |  |  |  |  | 2(4%) |  |
| Propanoate met | 4(2%) | 2(2%) |  |  |  |  | 3(2%) | 2(2%) |  |  |  |
| Pyrimidine met |  | 3(2%) |  |  | 4 (4%) |  |  | 2(2%) |  |  |  |
| Pyruvate met | 5(3%) | 2(2%) |  | 2(2%) |  |  | 4(3%) | 2(2%) |  |  |  |
| Purine met | 3(2%) | 5(4%) |  | 2(2%) | 4 (4%) |  |  | 2(2%) |  |  |  |
| Ribosome |  | 17(16%) |  |  | 5 (5%) |  |  | 9(9%) |  |  |  |
| RNA polymerase |  | 2(2%) |  |  |  |  |  |  |  |  |  |
| RNA degradation | 2(1%) | 2(2%) |  |  |  |  |  |  |  | 2(4%) |  |
| Starch and sucrose met | 11 (6%) |  |  | 4(3%) | 3 (3%) |  |  | 6(6%) |  | 3(6%) | 6(11%) |
| Thiamine met | 2(1%) |  |  |  |  |  |  |  |  |  |  |
| Tyrosine met | 2(1%) |  |  |  |  |  |  |  |  |  |  |
| Terpenoid backbone | 3(2%) |  |  | 2(2%) |  |  | 5 (2%) |  |  |  |  |
| Valine, leucine and isoleucine biosynthesis | 4(2%) |  |  |  |  |  | 2(1%) |  |  |  |  |
| Others | 10(5%) | 14(14%) |  | 26(21%) | 17(17%) |  | 24(15%) | 17(17%) |  | 18(33%) | 17(30%) |

**Table S5. Pathways differently regulated during *in vivo* *S. suis* infection.** The amount of genes regulated within a certain pathway, according to KEGG database, is shown for different infection sites. The percentage of differentially expressed genes is also indicated. Only statistically significant expressed genes (*p* < 0.01) homologous to P1/7 genome sequences and with at least ≤ -2 or ≥ 2 fold change differences relative to genes expressed during growth in THB were considered. Pathways with only one regulated gene were grouped as others. Down and Up, downregulated and upregulated, respectively; PTS, Phosphotransferase systems; met, metabolism.
